# Supplementary material for: Associations between low Apgar scores and mortality by race in the United States: A cohort study of 6,809,653 infants
Source: PLoS Med. 2022 Jul 12;19(7):e1004040. doi: 10.1371/journal.pmed.1004040 (PMC9275714; doi:10.1371/journal.pmed.1004040)
Supplement: S2 Table — (DOCX) [file pmed.1004040.s002.docx]

**Supplementary Table 2: Maternal Race Recategorizations**

| **Self-Reported Race on Birth Certificate** | **Race Category for this Analysis** |
| --- | --- |
| White | Non-Hispanic White |
| Black | Non-Hispanic Black |
| Asian Indian | Non-Hispanic Asian |
| Chinese |  |
| Japanese |  |
| Korean |  |
| Vietnamese |  |
| Other Asian |  |
| Mexican, Mexican American, Chicana | Hispanic |
| Puerto Rican |  |
| Cuban |  |
| Other Hispanic |  |
| Native Hawaiian | Non-Hispanic Other |
| Filipino |  |
| Guamanian or Chamorro |  |
| Samoan |  |
| Other Pacific Islander |  |
| American Indian or Alaskan Native |  |
| Other |  |
