# Supplementary material for: Associations between low Apgar scores and mortality by race in the United States: A cohort study of 6,809,653 infants
Source: PLoS Med. 2022 Jul 12;19(7):e1004040. doi: 10.1371/journal.pmed.1004040 (PMC9275714; doi:10.1371/journal.pmed.1004040)
Supplement: S3 Table — (DOCX) [file pmed.1004040.s003.docx]

**Supplementary Table 3: Mortality rates per 1,000 births, stratified by Apgar score and maternal race**

| **Racial Group** | **Apgar score category** | **% of total group**  **(95% CI)** | **Early neonatal mortality rate per 1,000 births (95% CI)** | **P-value*** | **Overall neonatal mortality rate per 1,000 births (95% CI)** | **P-value*** | **Infant mortality rate per 1,000 births (95% CI)** | **P-value*** |
| --- | --- | --- | --- | --- | --- | --- | --- | --- |
| **Non-Hispanic White** | Normal (n=3547228) | 98·74 (98·73-98·76) | 0·098 (0·089-0·11) | <0·001 | 0·30 (0·28-0·31) | <0·001 | 1·40 (1·36-1·44) | <0·001 |
|  | Intermediate (n=36144) | 1·02 (1·01-1·03) | 5·1 (4·4-5·9) |  | 7·7 (6·8-8·7) |  | 11·0 (9·9-12·08) |  |
|  | Low (n=8863) | 0·25 (0·24-0·252) | 63·6 (58·8-68·9) |  | 72·4 (67·2-78·04) |  | 79·3 (73·9-85·2) |  |
|  | Overall (n=3592235) |  | 0·31 (0·29-0·32) |  | 0·55 (0·52-0·57) |  | 1·69 (1·64-1·73) |  |
| **Hispanic** | Normal (n=1601375) | 99·18 (99·17-99·20) | 0·11 (0·09-0·13) | <0·001 | 0·27 (0·25-0·30) | <0·001 | 1·18 (1·13-1·23) | <0·001 |
|  | Intermediate (n=10124) | 0·63 (0·61-0·64) | 7·3 (5·8-9·2) |  | 10·6 (8·8-12·8) |  | 15·9 (13·6-18·5) |  |
|  | Low (n=3080) | 0·19 (0·18-0·20) | 66·6 (58·3-76·0) |  | 76·0 (67·2-85·9) |  | 87·7 (78·2-98·2) |  |
|  | Overall (n=1614579) |  | 0·28 (0·26-0·31) |  | 0·48 (0·45-0·51) |  | 1·44 (1·38-1·50) |  |
| **Non-Hispanic Black** | Normal (n=923131) | 98·32 (98·30-98·35) | 0·14 (0·12-0·16) | <0·001 | 0·44 (0·40-0·49) | <0·001 | 2·7 (2·6-2·8) | <0·001 |
|  | Intermediate (n=11816) | 1·26 (1·24-1·28) | 6·8 (5·5-8·5) |  | 10·4 (8·7-12·4) |  | 16·8 (14·7-19·3) |  |
|  | Low (n=3931) | 0·42 (0·41-0·43) | 45·8 (39·5-53·0) |  | 53·9 (47·3-61·5) |  | 62·3 (55·2-70·4) |  |
|  | Overall (n=938878) |  | 0·40 (0·37-0·45) |  | 0·8 (0·7-0·9) |  | 3·2 (3·04-3·3) |  |
| **Non-Hispanic Asian** | Normal (n=447842) | 99·18 (99·15-99·21) | 0·11 (0·09-0·15) | <0·001 | 0·22 (0·18-0·27) | <0·001 | 0·8 (0·7-0·9) | <0·001 |
|  | Intermediate (n=2919) | 0·65 (0·62-0·67) | 5·5 (3·4-8·9) |  | 7·5 (5·0-11·4) |  | 13·02 (9·5-17·9) |  |
|  | Low (n=785) | 0·17 (0·16-0·19) | 59·9 (45·4-79·0) |  | 71·3 (55·4-91·8) |  | 76·4 (59·9-97·5) |  |
|  | Overall (n=451546) |  | 0·25 (0·21-0·30) |  | 0·39 (0·33-0·45) |  | 1·0 (0·91-1·09) |  |
| **Non-Hispanic Other** | Normal (n=209253) | 98·51 (98·46-98·56) | 1·2 (0·8-1·8) | <0·001 | 0·42 (0·34-0·52) | <0·001 | 2·7 (2·5-3·0) | <0·001 |
|  | Intermediate (n=2464) | 1·16 (1·11-1·21) | 3·7 (1·9-7·01) |  | 6·5 (4·0-10·6) |  | 11·8 (8·2-16·9) |  |
|  | Low (n=698) | 0·33 (0·31-0·35) | 587·4 (436·5-790·5) |  | 65·9 (49·8-87·1) |  | 85·5 (66·4-110·1) |  |
|  | Overall (n=212415) |  | 0·35 (0·28-0·44) |  | 0·7 (0·6-0·8) |  | 3·09 (2·86-3·33) |  |

*Chi-square test for trend comparing mortality rates among Apgar score categories

*CI=Confidence Interval*
