# Supplementary material for: Associations between low Apgar scores and mortality by race in the United States: A cohort study of 6,809,653 infants
Source: PLoS Med. 2022 Jul 12;19(7):e1004040. doi: 10.1371/journal.pmed.1004040 (PMC9275714; doi:10.1371/journal.pmed.1004040)
Supplement: S4 Table — (DOCX) [file pmed.1004040.s004.docx]

**Supplementary Table 4: Unadjusted odds ratios for early neonatal, overall neonatal, and infant mortality by race group**

|  | **Early Neonatal Mortality**  **(0-6 days)** | | | **Overall Neonatal Mortality**  **(0-27 days)** | | | **Infant Mortality**  **(0-365 days)** | | |
| --- | --- | --- | --- | --- | --- | --- | --- | --- | --- |
| **Race** | **Mortality (Deaths per 1,000 births)** | **Unadjusted OR**  **(95% CI)** | **P-value*** | **Mortality (Deaths per 1,000 births)** | **Unadjusted OR**  **(95% CI)** | **P-value*** | **Mortality (Deaths per 1,000 births)** | **Unadjusted OR**  **(95% CI)** | **P-value*** |
| **NH White**  n=3592235 | 1096 (0.3) | 1 (ref) | - | 1967 (0.5) | 1 (ref) | - | 6051 (1.7) | 1 (ref) | - |
| **Hispanic**  n=1614579 | 451 (0.3) | 0.92 (0.82-1.02) | 0.11 | 772 (0.5) | 0.87 (0.80-0.95) | 0.001 | 2317 (1.4) | 0.85 (0.81-0.89) | <0.001 |
| **NH Black**  n=938878 | 379 (0.4) | 1.32 (1.18-1.49) | <0.001 | 746 (0.8) | 1.45 (1.33-1.58) | <0.001 | 2962 (3.2) | 1.88 (1.80-1.96) | <0.001 |
| **NH Asian**  n=451546 | 114 (0.3) | 0.83 (0.68-1.00) | 0.05 | 176 (0.4) | 0.71 (0.61-0.83) | <0.001 | 450 (1.0) | 0.59 (0.54-0.65) | <0.001 |
| **NH Other**  n=212415 | 75 (0.4) | 1.16 (0.92-1.46) | 0.22 | 150 (0.7) | 1.29 (1.09-1.52) | 0.003 | 656 (3.1) | 1.84 (1.69-1.99) | <0.001 |

*****Wald p-values

*NH = Non-Hispanic, OR = Odds Ratio, CI = Confidence Interval*
