# Supplementary material for: Associations between low Apgar scores and mortality by race in the United States: A cohort study of 6,809,653 infants
Source: PLoS Med. 2022 Jul 12;19(7):e1004040. doi: 10.1371/journal.pmed.1004040 (PMC9275714; doi:10.1371/journal.pmed.1004040)
Supplement: S5 Table — (DOCX) [file pmed.1004040.s005.docx]

**Supplementary Table 5: Unadjusted and Adjusted Odds Ratios for Mortality for Multivariable Models in Non-Hispanic White Cohort**

|  | **Early neonatal mortality (0-6 days)** | | | **Overall Neonatal Mortality (<28 days)** | | | **Infant Mortality (<1 year)** | | |
| --- | --- | --- | --- | --- | --- | --- | --- | --- | --- |
|  | Early neonatal mortality [n (deaths per 1,000 births)] | Unadjusted OR (95% CI) | Adjusted OR (95% CI) | Overall neonatal mortality [n (deaths per 1,000 births)] | Unadjusted OR (95% CI) | Adjusted OR (95% CI) | Infant mortality [n (deaths per 1,000 births)] | Unadjusted OR (95% CI) | Adjusted OR (95% CI) |
| **5-Minute Apgar** |  |  |  |  |  |  |  |  |  |
| Normal (7-10) | 349 (0.1) | 1 | 1 | 1047 (0.3) | 1 | 1 | 4952 (1.4) | 1 | 1 |
| Intermediate (4-6) | 183 (5.1) | 51.7 (43.2-61.9) | 44.4 (37.0-53.4)** | 278 (7.7) | 26.3 (23.0-30.0) | 22.0 (19.2-25.3)** | 396 (11.0) | 7.9 (7.2-8.8) | 6.9 (6.2-7.6)** |
| Low (0-3) | 564 (63.6) | 690.7 (603.3-790.7) | 598.9 (519.9-690.0)** | 642 (72.4) | 264.5 (239.2-292.5) | 225.0 (202.4-250.2)** | 703 (79.3) | 61.6 (56.8-66.9) | 54.4 (49.9-59.4)** |
| **Year of birth** |  |  |  |  |  |  |  |  |  |
| 2016 | 562 (0.3) | 1 | 1 | 981 (0.5) | 1 | 1 | 3080 (1.7) | 1 | 1 |
| 2017 | 534 (0.3) | 0.98 (0.87-1.10) | 0.98 (0.87-1.11) | 986 (0.6) | 1.04 (0.95-1.13) | 1.04 (0.95-1.14) | 2971 (1.7) | 0.99 (0.95-1.05) | 1.00 (0.95-1.06) |
| **Infant Sex** |  |  |  |  |  |  |  |  |  |
| Male | 561 (0.3) | 1 | 1 | 1025 (0.6) | 1 | 1 | 3344 (1.8) | 1 | 1 |
| Female | 535 (0.3) | 0.99 (0.88-1.12) | 1.09 (0.96-1.23) | 942 (0.5) | 0.96 (0.88-1.05) | 0.98 (0.89-1.07) | 2707 (1.5) | 0.84 (0.80-0.89) | 0.81 (0.76-0.85)** |
| **Smoking Status** |  |  |  |  |  |  |  |  |  |
| No | 903 (0.3) | 1 | 1 | 1540 (0.5) | 1 | 1 | 4351 (1.3) | 1 | 1 |
| Yes | 177 (0.5) | 1.8 (1.5-2.1) | 1.1 (0.9-1.3) | 402 (1.1) | 2.4 (2.1-2.7) | 1.5 (1.3-1.7)** | 1636 (4.6) | 3.4 (3.2-3.6) | 1.9 (1.8-2.0)** |
| Unknown | 16 (1.1) | 3.9 (2.4-6.4) | 1.7 (1.0-3.1) | 25 (1.7) | 3.6 (2.4-5.3) | 2.0 (1.3-3.0)* | 64 (4.4) | 3.3 (2.6-4.2) | 2.0 (1.6-2.6)** |
| **Birthweight (g)** |  |  |  |  |  |  |  |  |  |
| 2000-2499 | 119 (1.8) | 1 | 1 | 203 (3.1) | 1 | 1 | 476 (7.3) | 1 | 1 |
| <1500 | 21 (39.4) | 22.3 (13.9-35.8) | 7.4 (3.9-14.0)** | 26 (48.8) | 16.3 (10.8-24.8) | 7.2 (4.2-12.4)** | 36 (67.5) | 9.8 (6.9-13.9) | 6.7 (4.5-10.1)** |
| 1500-1999 | 65 (16.4) | 9.1 (6.7-12.3) | 5.3 (3.7-7.7)** | 82 (20.6) | 6.7 (5.2-8.7) | 4.3 (3.2-5.8)** | 130 (32.7) | 4.6 (3.8-5.6) | 3.5 (2.9-4.4)** |
| 2500-2999 | 259 (0.5) | 0.28 (0.23-0.35) | 0.44 (0.35-0.55)** | 496 (1.0) | 0.32 (0.27-0.37) | 0.48 (0.38-0.54)** | 1457 (2.9) | 0.39 (0.36-0.44) | 0.51 (0.46-0.57)** |
| 3000-3499 | 323 (0.2) | 0.12 (0.10-0.15) | 0.24 (0.19-0.30)** | 629 (0.4) | 0.14 (0.12-0.16) | 0.25 (0.21-0.29)** | 2206 (1.5) | 0.21 (0.19-0.23) | 0.32 (0.29-0.36)** |
| 3500-3999 | 208 (0.2) | 0.10 (0.08-0.12) | 0.20 (0.15-0.26)** | 381 (0.3) | 0.10 (0.09-0.12) | 0.2 (0.16-0.24)** | 1303 (1.1) | 0.15 (0.13-0.17) | 0.25 (0.22-0.28)** |
| 4000-4499 | 75 (0.2) | 0.12 (0.09-0.16) | 0.23 (0.17-0.32)** | 115 (0.3) | 0.11 (0.09-0.14) | 0.2 (0.16-0.26)** | 374 (1.1) | 0.15 (0.13-0.17) | 0.26 (0.22-0.3)** |
| 4500-4999 | 17 (0.4) | 0.20 (0.12-0.33) | 0.26 (0.15-0.45)** | 23 (0.5) | 0.16 (0.10-0.24) | 0.22 (0.14-0.34)** | 52 (1.1) | 0.15 (0.11-0.20) | 0.22 (0.16-0.29)** |
| >5000 | 9 (1.9) | 1.02 (0.5-2.00) | 0.8 (0.4-1.7) | 12 (0.5) | 0.8 (0.4-1.4) | 0.8 (0.4-1.4) | 15 (3.1) | 0.4 (0.3-0.7) | 0.5 (0.3-0.8)* |
| Unknown | 0 (0) | -- | -- | 0 (0) | -- | -- | 2 (1.4) | 0.2 (0.1-0.7) | 0.2 (0.04-0.7)* |
| **Maternal Education** |  |  |  |  |  |  |  |  |  |
| <8th grade | 40 (0.9) | 1 | 1 | 67 (1.5) | 1 | 1 | 167 (3.7) | 1 | 1 |
| 9-12th grade, no diploma | 102 (0.5) | 0.6 (0.4-0.8) | 0.5 (0.4-0.8)* | 203 (1.0) | 0.7 (0.5-0.9) | 0.6 (0.4-0.8)** | 862 (4.1) | 1.1 (1.0-1.3) | 0.9 (0.7-1.04) |
| HS or GED | 244 (0.3) | 0.4 (0.3-0.5) | 0.4 (0.3-0.6)** | 505 (0.7) | 0.5 (0.4-0.6) | 0.5 (0.4-0.6)** | 1919 (2.6) | 0.7 (0.6-0.8) | 0.7 (0.6-0.9)** |
| Some college credit | 249 (0.3) | 0.4 (0.3-0.5) | 0.5 (0.3-0.7)** | 447 (0.6) | 0.4 (0.3-0.5) | 0.5 (0.4-0.7)** | 1407 (1.9) | 0.53 (0.45-0.62) | 0.7 (0.6-0.8)** |
| Associates Degree | 83 (0.2) | 0.3 (0.2-0.4) | 0.4 (0.3-0.6)** | 159 (0.5) | 0.3 (0.2-0.4) | 0.4 (0.3-0.6)** | 433 (1.2) | 0.34 (0.28-0.40) | 0.5 (0.4-0.6)** |
| Bachelors Degree | 220 (0.2) | 0.3 (0.2-0.4) | 0.4 (0.3-0.6)** | 336 (0.4) | 0.24 (0.18-0.31) | 0.4 (0.3-0.5)** | 767 (0.8) | 0.22 (0.19-0.26) | 0.4 (0.3-0.5)** |
| Masters Degree | 95 (0.2) | 0.3 (0.2-0.4) | 0.4 (0.3-0.7)** | 162 (0.4) | 0.25 (0.19-0.34) | 0.4 (0.3-0.6)** | 315 (0.7) | 0.20 (0.16-0.24) | 0.4 (0.3-0.5)** |
| Doctorate/Professional Degree | 26 (0.2) | 0.24 (0.15-0.40) | 0.4 (0.3-0.7)* | 46 (0.4) | 0.3 (0.2-0.4) | 0.5 (0.3-0.7)** | 110 (0.9) | 0.25 (0.19-0.31) | 0.5 (0.4-0.7)** |
| Unknown | 37 (2.1) | 2.4 (1.5-3.8) | 1.9 (1.2-3.3)* | 42 (2.4) | 1.6 (1.1-2.4) | 1.4 (0.9-2.2) | 71 (4.1) | 1.1 (0.8-1.5) | 1.2 (0.9-1.6) |
| **Maternal BMI** |  |  |  |  |  |  |  |  |  |
| Underweight (<18.5) | 35 (0.3) | 1 | 1 | 71 (0.6) | 1 | 1 | 238 (2.0) | 1 | 1 |
| Normal (18.5-24.9) | 445 (0.3) | 0.9 (0.6-1.2) | 1.1 (0.8-1.6) | 815 (0.5) | 0.8 (0.6-1.0) | 1.1 (0.9-1.4) | 2524 (1.5) | 0.7 (0.6-0.8) | 1.2 (1.02-1.3)* |
| Overweight (25-29.9) | 284 (0.3) | 1.1 (0.8-1.5) | 1.3 (0.9-1.9) | 494 (0.6) | 0.9 (0.7-1.2) | 1.3 (1.0-1.7) | 1399 (1.6) | 0.8 (0.7-0.9) | 1.2 (1.1-1.4)* |
| Obesity I (30-34.9) | 144 (0.3) | 1.1 (0.7-1.5) | 1.2 (0.8-1.7) | 271 (0.6) | 1.0 (0.8-1.3) | 1.2 (0.9-1.6) | 924 (2.0) | 1.0 (0.9-1.1) | 1.4 (1.2-1.7)** |
| Obesity II (35-39.9) | 80 (0.3) | 1.2 (0.8-1.7) | 1.2 (0.8-1.8) | 139 (0.6) | 1.0 (0.8-1.3) | 1.2 (0.9-1.6) | 441 (1.9) | 0.9 (0.8-1.1) | 1.3 (1.1-1.6)** |
| Obesity III (>40) | 62 (0.4) | 1.3 (0.9-1.96) | 1.1 (0.7-1.7) | 109 (0.7) | 1.1 (0.8-1.5) | 1.2 (0.9-1.6) | 347 (2.2) | 1.1 (0.9-1.3) | 1.4 (1.2-1.7)** |
| Unknown | 46 (0.7) | 2.2 (1.4-3.5) | 1.7 (1.1-2.8) | 68 (1.0) | 1.6 (1.2-2.3) | 1.6 (1.1-2.3) | 178 (2.6) | 1.3 (1.0-1.5) | 1.6 (1.3-2.0)** |
| **Maternal age** |  |  |  |  |  |  |  |  |  |
| 15-19 | 49 (0.4) | 1 | 1 | 117 (0.9) | 1 | 1 | 477 (3.6) | 1 | 1 |
| 20-24 | 228 (0.4) | 1.0 (0.7-1.3) | 1.2 (0.9-1.7) | 447 (0.7) | 0.8 (0.7-1.0) | 0.9 (0.8-1.2) | 1656 (2.6) | 0.73 (0.66-0.81) | 0.8 (0.7-0.9)** |
| 25-29 | 310 (0.3) | 0.8 (0.6-1.1) | 1.2 (0.8-1.7) | 562 (0.5) | 0.6 (0.5-0.7) | 0.8 (0.7-1.1) | 1763 (1.6) | 0.45 (0.41-0.50) | 0.57 (0.50-0.64)** |
| 30-34 | 307 (0.3) | 0.7 (0.6-1.0) | 1.2 (0.8-1.7) | 529 (0.5) | 0.5 (0.4-0.7) | 0.8 (0.7-1.1) | 1411 (1.3) | 0.35 (0.31-0.39) | 0.51 (0.45-0.57)** |
| 35-39 | 160 (0.3) | 0.9 (0.6-1.2) | 1.2 (0.8-1.8) | 241 (0.5) | 0.5 (0.4-0.7) | 0.8 (0.6-1.0)* | 593 (1.2) | 0.32 (0.29-0.36) | 0.44 (0.38-0.51)** |
| 40+ | 42 (0.4) | 1.2 (0.8-1.8) | 1.5 (0.9-2.3) | 71 (0.7) | 0.8 (0.6-1.1) | 1.0 (0.7-1.4) | 151 (1.6) | 0.44 (0.36-0.52) | 0.5 (0.4-0.6)** |
| **Previous live births** |  |  |  |  |  |  |  |  |  |
| 1 to 2 | 453 (0.3) | 1 | 1 | 849 (0.5) | 1 | 1 | 2997 (1.7) | 1 | 1 |
| None | 491 (0.3) | 1.4 (1.2-1.5) | 0.8 (0.7-1.0)* | 850 (0.6) | 1.2 (1.1-1.4) | 0.9 (0.8-1.0)* | 2099 (1.5) | 0.88 (0.83-0.93) | 0.69 (0.65-0.73)** |
| 3 to 4 | 106 (0.4) | 1.4 (1.2-1.8) | 1.1 (0.9-1.4) | 190 (0.6) | 1.4 (1.2-1.6) | 1.1 (0.9-1.3) | 746 (2.5) | 1.5 (1.4-1.6) | 1.3 (1.2-1.4)** |
| 5 or more | 34 (0.5) | 2.0 (1.4-2.9) | 1.2 (0.8-1.7) | 62 (0.9) | 2.0 (1.5-2.6) | 1.4 (1.0-1.8) | 180 (2.7) | 1.6 (1.4-1.9) | 1.4 (1.2-1.7)** |
| Unknown | 12 (1.2) | 4.8 (2.7-8.6) | 1.7 (0.9-3.3) | 16 (1.6) | 3.4 (2.1-5.6) | 1.6 (1.0-2.8) | 29 (2.9) | 1.8 (1.2-2.5) | 1.2 (0.8-1.7) |
| **Gestational age [mean (SD)]** | 38.68 (1.3) | 0.70 (0.67-0.74) | 0.92 (0.87-0.97)* | 38.66 (1.25) | 0.70 (0.67-0.73) | 0.90 (0.86-0.94)** | 38.73 (1.14) | 0.74 (0.72-0.75) | 0.91 (0.89-0.93)** |

*Wald p-value < 0.05; **Wald p-value < 0.001

*OR (95% CI)= Odds ratios and associated 95% confidence intervals; GED=General Educational Development; BMI=Body Mass Index; SD=Standard Deviation*

Odds ratios and 95% confidence intervals were adjusted for infant sex, maternal age, maternal smoking status, infant birthweight, maternal education, maternal BMI, previous number of live births and gestational age.
