# Supplementary material for: Associations between low Apgar scores and mortality by race in the United States: A cohort study of 6,809,653 infants
Source: PLoS Med. 2022 Jul 12;19(7):e1004040. doi: 10.1371/journal.pmed.1004040 (PMC9275714; doi:10.1371/journal.pmed.1004040)
Supplement: S6 Table — (DOCX) [file pmed.1004040.s006.docx]

**Supplementary Table 6: Unadjusted and Adjusted Odds Ratios for Mortality for Multivariable Models in Hispanic Cohort**

|  | **Early Neonatal Mortality (<7 days)** | | | **Overall Neonatal Mortality (<28 days)** | | | **Infant Mortality (<1 year)** | | |
| --- | --- | --- | --- | --- | --- | --- | --- | --- | --- |
|  | Early neonatal mortality [n (deaths per 1,000 births)] | Unadjusted OR (95% CI) | Adjusted OR (95% CI) | Overall neonatal mortality [n (deaths per 1,000 births)] | Unadjusted OR (95% CI) | Adjusted OR (95% CI) | Infant mortality [n (deaths per 1,000 births)] | Unadjusted OR (95% CI) | Adjusted OR (95% CI) |
| **5-Minute Apgar** |  |  |  |  |  |  |  |  |  |
| Normal (7-10) | 172 (0.1) | 1 | 1 | 431 (0.3) | 1 | 1 | 1886 (1.2) | 1 | 1 |
| Intermediate (4-6) | 74 (7.3) | 68.6 (52.2-90.1) | 59.0 (44.5-78.2)** | 107 (10.6) | 39.7 (32.1-49.1) | 33.2 (26.7-41.4)** | 161 (15.9) | 13.7 (11.7-16.1) | 12.4 (10.5-14.7)** |
| Low (0-3) | 205 (66.6) | 663.8 (540.3-815.6) | 537.1 (431.8-668.2)** | 234 (76.0) | 305.4 (259.4-359.6) | 245.1 (205.8-291.9)** | 270 (87.7) | 81.5 (71.4-93.1) | 70.02 (60.8-80.7)** |
| **Year of birth** |  |  |  |  |  |  |  |  |  |
| 2016 | 223 (0.3) | 1 | 1 | 393 (0.5) | 1 | 1 | 1168 (1.4) | 1 | 1 |
| 2017 | 228 (0.3) | 1.1 (0.9-1.3) | 1.1 (0.9-1.3) | 379 (0.5) | 1.0 (0.9-1.1) | 1.0 (0.9-1.2) | 1149 (1.4) | 1.01 (0.9-1.1) | 1.02 (0.9-1.1) |
| **Infant Sex** |  |  |  |  |  |  |  |  |  |
| Male | 247 (0.3) | 1 | 1 | 425 (0.5) | 1 | 1 | 1255 (1.5) | 1 | 1 |
| Female | 204 (0.3) | 0.9 (0.7-1.02) | 0.8 (0.7-1.01) | 347 (0.4) | 0.8 (0.7-1.0) | 0.8 (0.7-0.9)* | 1062 (1.3) | 0.87 (0.80-0.94) | 0.82 (0.76-0.89)** |
| **Smoking Status** |  |  |  |  |  |  |  |  |  |
| No | 434 (0.3) | 1 | 1 | 739 (0.5) | 1 | 1 | 2198 (1.4) | 1 | 1 |
| Yes | 12 (0.4) | 1.6 (0.9-2.9) | 1.0 (0.6-1.8) | 26 (1.0) | 2.1 (1.4-3.1) | 1.4 (0.9-2.1) | 105 (3.9) | 2.8 (2.3-3.4) | 2.0 (1.6-2.4)** |
| Unknown | 5 (0.8) | 2.8 (1.2-6.7) | 1.7 (0.6-4.5) | 7 (1.1) | 2.3 (1.1-4.8) | 1.5 (0.7-3.4) | 14 (2.1) | 1.5 (0.9-2.6) | 1.2 (0.7-2.1) |
| **Birthweight (g)** |  |  |  |  |  |  |  |  |  |
| 2000-2499 | 77 (2.3) | 1 | 1 | 120 (3.6) | 1 | 1 | 234 (7.1) | 1 | 1 |
| <1500 | 9 (31.7) | 14.0 (7.0-28.2) | 5.6 (2.2-14.6)** | 11 (38.7) | 11.1 (5.9-20.7) | 5.4 (2.4-12.2)** | 20 (70.4) | 10.6 (6.6-17.03) | 7.8 (4.5-13.6)** |
| 1500-1999 | 34 (19.5) | 8.5 (5.7-12.8) | 3.7 (2.2-6.2)** | 48 (27.6) | 7.8 (5.5-10.9) | 4.3 (2.8-6.5)** | 82 (47.1) | 6.9 (5.4-9.0) | 5.1 (3.8-6.8)** |
| 2500-2999 | 115 (0.4) | 0.2 (0.1-0.2) | 0.3 (0.2-0.4)** | 202 (0.7) | 0.20 (0.16-0.25) | 0.3 (0.2-0.4)** | 577 (2.1) | 0.29 (0.25-0.34) | 0.34 (0.29-0.40)** |
| 3000-3499 | 122 (0.2) | 0.07 (0.06-0.10) | 0.14 (0.10-0.19)** | 213 (0.3) | 0.08 (0.07-0.10) | 0.14 (0.11-0.18)** | 827 (1.2) | 0.16 (0.14-0.19) | 0.21 (0.18-0.25)** |
| 3500-3999 | 66 (0.1) | 0.06 (0.04-0.08) | 0.10 (0.07-0.15)** | 130 (0.3) | 0.08 (0.06-0.10) | 0.12 (0.09-0.16)** | 461 (1.0) | 0.14 (0.12-0.16) | 0.18 (0.15-0.22)** |
| 4000-4499 | 23 (0.2) | 0.09 (0.06-0.14) | 0.13 (0.08-0.21)** | 38 (0.4) | 0.10 (0.07-0.14) | 0.14 (0.09-0.20)** | 97 (0.9) | 0.13 (0.10-0.16) | 0.15 (0.12-0.20)** |
| 4500-4999 | 4 (0.3) | 0.1 (0.04-0.3) | 0.09 (0.03-0.25)** | 9 (0.6) | 0.16 (0.08-0.32) | 0.2 (0.1-0.3)** | 16 (1.1) | 0.15 (0.09-0.25) | 0.15 (0.09-0.25)** |
| >5000 | 1 (0.5) | 0.2 (0.03-1.5) | 0.1 (0.02-0.9)* | 1 (0.5) | 0.13 (0.02-0.96) | 0.1 (0.01-0.6)* | 1 (0.5) | 0.1 (0.01-0.5) | 0.05 (0.01-0.35)* |
| Unknown | 0 (0) | -- | -- | 0 (0) | -- | -- | 2 (7.6) | 1.1 (0.3-4.4) | 0.8 (0.2-3.4) |
| **Maternal Education** |  |  |  |  |  |  |  |  |  |
| <8th grade | 47 (0.3) | 1 | 1 | 75 (0.5) | 1 | 1 | 226 (1.5) | 1 | 1 |
| 9-12th grade, no diploma | 104 (0.3) | 1.1 (0.8-1.5) | 1.1 (0.8-1.6) | 177 (0.6) | 1.2 (0.9-1.5) | 1.1 (0.8-1.5) | 574 (1.9) | 1.3 (1.1-1.5) | 1.1 (0.9-1.3) |
| HS or GED | 149 (0.3) | 0.9 (0.7-1.3) | 1.0 (0.7-1.4) | 261 (0.5) | 1.02 (0.8-1.3) | 1.0 (0.8-1.3) | 783 (1.5) | 1.01 (0.9-1.2) | 1.0 (0.8-1.1) |
| Some college credit | 65 (0.2) | 0.7 (0.5-1.0) | 0.7 (0.5-1.1) | 126 (0.4) | 0.8 (0.6-1.1) | 0.8 (0.6-1.1) | 418 (1.3) | 0.9 (0.8-1.03) | 0.9 (0.8-1.1) |
| Associates Degree | 19 (0.2) | 0.6 (0.4-1.1) | 0.6 (0.3-1.1) | 35 (0.4) | 0.7 (0.5-1.1) | 0.7 (0.5-1.1) | 95 (1.0) | 0.7 (0.5-0.8) | 0.7 (0.6-0.9)* |
| Bachelors Degree | 36 (0.2) | 0.8 (0.5-1.2) | 0.9 (0.5-1.4) | 54 (0.4) | 0.7 (0.5-1.02) | 0.8 (0.6-1.2) | 131 (0.9) | 0.6 (0.5-0.7) | 0.8 (0.6-1.0)* |
| Masters Degree | 16 (0.3) | 1.1 (0.6-1.9) | 1.4 (0.8-2.5) | 22 (0.5) | 0.9 (0.6-1.5) | 1.2 (0.7-1.9) | 44 (0.9) | 0.6 (0.5-0.9) | 1.0 (0.7-1.3) |
| Doctorate/Professional Degree | 2 (0.2) | 0.5 (0.1-2.1) | 0.6 (0.1-2.4) | 4 (0.3) | 0.7 (0.2-1.8) | 0.7 (0.3-2.01) | 8 (0.6) | 0.4 (0.2-0.9) | 0.6 (0.3-1.3) |
| Unknown | 13 (0.6) | 2.0 (1.1-3.7) | 1.4 (0.7-2.8) | 18 (0.9) | 1.7 (1.03-2.9) | 1.3 (0.8-2.3) | 38 (1.8) | 1.2 (0.9-1.7) | 1.1 (0.8-1.6) |
| **Maternal BMI** |  |  |  |  |  |  |  |  |  |
| Underweight (<18.5) | 4 (0.1) | 1 | 1 | 17 (0.4) | 1 | 1 | 64 (1.6) | 1 | 1.00 |
| Normal (18.5-24.9) | 168 (0.3) | 2.8 (1.04-7.6) | 3.5 (1.3-9.5)* | 276 (0.5) | 1.1 (0.7-1.8) | 1.4 (0.8-2.3) | 802 (1.3) | 0.8 (0.7-1.1) | 1.1 (0.8-1.4) |
| Overweight (25-29.9) | 120 (0.3) | 2.5 (0.9-6.9) | 3.2 (1.2-8.8)* | 210 (0.4) | 1.04 (0.6-1.7) | 1.4 (0.8-2.3) | 677 (1.4) | 0.9 (0.7-1.2) | 1.2 (1.0-1.6) |
| Obesity I (30-34.9) | 71 (0.3) | 2.6 (1.0-7.2) | 3.4 (1.2-9.6)* | 125 (0.5) | 1.1 (0.7-1.8) | 1.5 (0.9-2.5) | 381 (1.4) | 0.9 (0.7-1.2) | 1.2 (0.9-1.6) |
| Obesity II (35-39.9) | 37 (0.3) | 3.2 (1.1-8.9) | 4.3 (1.5-12.3)* | 61 (0.5) | 1.2 (0.7-2.1) | 1.7 (1.0-3.0) | 172 (1.5) | 0.9 (0.7-1.2) | 1.3 (1.0-1.8) |
| Obesity III (>40) | 22 (0.3) | 3.2 (1.1-9.3) | 3.6 (1.2-10.5)* | 41 (0.6) | 1.4 (0.8-2.5) | 1.7 (0.9-3.0) | 135 (2.0) | 1.2 (0.9-1.7) | 1.7 (1.2-2.3)* |
| Unknown | 29 (0.6) | 6.5 (2.3-18.4) | 5.8 (2.0-16.9)* | 42 (0.9) | 2.2 (1.3-3.9) | 2.2 (1.2-4.0)* | 86 (1.9) | 1.2 (0.9-1.7) | 1.4 (1.01-2.0)* |
| **Maternal age** |  |  |  |  |  |  |  |  |  |
| 15-19 | 50 (0.4) | 1 | 1 | 89 (0.7) | 1 | 1 | 314 (2.4) | 1 | 1 |
| 20-24 | 103 (0.3) | 0.7 (0.5-0.9) | 0.8 (0.6-1.2) | 197 (0.5) | 0.7 (0.6-0.9) | 0.8 (0.6-1.1) | 672 (1.7) | 0.7 (0.6-0.8) | 0.7 (0.6-0.8)** |
| 25-29 | 105 (0.2) | 0.6 (0.4-0.8) | 0.8 (0.6-1.2) | 195 (0.4) | 0.6 (0.5-0.8) | 0.8 (0.6-1.1) | 618 (1.3) | 0.55 (0.48-0.63) | 0.53 (0.45-0.62)** |
| 30-34 | 97 (0.3) | 0.7 (0.5-1.0) | 0.9 (0.6-1.4) | 155 (0.4) | 0.6 (0.5-0.8) | 0.8 (0.6-1.1) | 415 (1.1) | 0.46 (0.40-0.54) | 0.43 (0.36-0.51)** |
| 35-39 | 71 (0.4) | 0.9 (0.6-1.3) | 1.2 (0.8-1.8) | 101 (0.5) | 0.7 (0.6-1.0) | 0.9 (0.6-1.2) | 229 (1.1) | 0.5 (0.4-0.6) | 0.39 (0.32-0.48)** |
| 40+ | 25 (0.5) | 1.3 (0.8-2.1) | 1.2 (0.7-2.1) | 35 (0.7) | 1.01 (0.7-1.5) | 0.9 (0.6-1.5) | 69 (1.4) | 0.6 (0.4-0.7) | 0.4 (0.3-0.5)** |
| **Previous live births** |  |  |  |  |  |  |  |  |  |
| 1 to 2 | 195 (0.2) | 1 | 1 | 345 (0.4) | 1 | 1 | 1111 (1.4) | 1 | 1 |
| None | 177 (0.3) | 1.3 (1.1-1.6) | 0.8 (0.6-1.03) | 302 (0.5) | 1.3 (1.1-1.5) | 0.9 (0.7-1.03) | 782 (1.4) | 1.03 (0.9-1.1) | 0.7 (0.6-0.8)** |
| 3 to 4 | 60 (0.3) | 1.2 (0.9-1.6) | 0.9 (0.6-1.2) | 98 (0.5) | 1.1 (0.9-1.4) | 0.9 ( 0.7-1.2) | 315 (1.5) | 1.1 (1.0-1.2) | 1.1 (1.0-1.3) |
| 5 or more | 18 (0.5) | 1.9 (1.2-3.1) | 1.1 (0.6-1.8) | 25 (0.6) | 1.5 (1.01-2.3) | 1.01 (0.6-1.6) | 105 (2.7) | 2.0 (1.6-2.4) | 2.03 (1.6-2.5)** |
| Unknown | 1 (0.3% | 1.3 (0.2-9.01) | 0.2 (0.02-1.7) | 2 (0.6) | 1.4 (0.4-5.7) | 0.4 (0.1-1.7) | 4 (1.2) | 0.9 (0.3-2.4) | 0.5 (0.2-1.3) |
| **Gestational age [mean(SD)]** | 38.5 (1.3) | 0.64 (0.59-0.70) | 0.9 (0.8-1.0)* | 38.5 (1.2) | 0.64 (0.60-0.69) | 0.84 (0.79-0.91)** | 38.6 (1.1) | 0.74 (0.71-0.77) | 0.90 (0.86-0.94)** |

*Wald p-value < 0.05; **Wald p-value < 0.001

*OR (95% CI)= Odds ratios and associated 95% confidence intervals; GED=General Educational Development; BMI=Body Mass Index; SD=Standard Deviation*

Odds ratios and 95% confidence intervals were adjusted for infant sex, maternal age, maternal smoking status, infant birthweight, maternal education, maternal BMI, previous number of live births and gestational age.
