# Supplementary material for: Associations between low Apgar scores and mortality by race in the United States: A cohort study of 6,809,653 infants
Source: PLoS Med. 2022 Jul 12;19(7):e1004040. doi: 10.1371/journal.pmed.1004040 (PMC9275714; doi:10.1371/journal.pmed.1004040)
Supplement: S7 Table — (DOCX) [file pmed.1004040.s007.docx]

**Supplementary Table 7: Unadjusted and Adjusted Odds Ratios for Mortality for Multivariable Models in Non-Hispanic Black Cohort**

|  | **Early Neonatal Mortality (<7 days)** | | | **Overall Neonatal Mortality (<28 days)** | | | **Infant Mortality (<1 year)** | | |
| --- | --- | --- | --- | --- | --- | --- | --- | --- | --- |
|  | Early neonatal mortality [n (deaths per 1,000 births)] | Unadjusted OR (95% CI) | Adjusted OR (95% CI) | Overall neonatal mortality [n (deaths per 1,000 births)] | Unadjusted OR (95% CI) | Adjusted OR (95% CI) | Infant mortality [n (deaths per 1,000 births)] | Unadjusted OR (95% CI) | Adjusted OR (95% CI) |
| **5-Minute Apgar** |  |  |  |  |  |  |  |  |  |
| Normal (7-10) | 127 (0.1) | 1 | 1 | 411 (0.4) | 1 | 1 | 2518 (2.7) | 1 | 1 |
| Intermediate (4-6) | 80 (6.8) | 49.5 (37.4-65.6) | 41.4 (31.1-55.3)** | 123 (10.4) | 23.6 (19.3-28.9) | 20.2 (16.4-24.9)** | 199 (16.8) | 6.3 (5.4-7.2) | 5.8 (5.0-6.8)** |
| Low (0-3) | 172 (43.8) | 332.6 (263.8-419.2) | 286.7 (225.6-364.3)** | 212 (53.9) | 128.0 (108.1-151.5) | 114.2 (95.7-136.1)** | 245 (62.3) | 24.3 (21.2-27.8) | 23.3 (20.3-26.8)** |
| **Year of birth** |  |  |  |  |  |  |  |  |  |
| 2016 | 194 (0.4) | 1 | 1 | 386 (0.8) | 1 | 1 | 1492 (3.2) | 1 | 1 |
| 2017 | 185 (0.4) | 1.0 (0.8-1.2) | 0.9 (0.8-1.2) | 360 (0.8) | 0.9 (0.8-1.1) | 0.9 (0.8-1.1) | 1470 (3.1) | 1.0 (0.9-1.1) | 1.0 (0.9-1.1) |
| **Infant Sex** |  |  |  |  |  |  |  |  |  |
| Male | 210 (0.4) | 1 | 1 | 403 (0.8) | 1 | 1 | 1568 (3.3) | 1 | 1 |
| Female | 169 (0.4) | 0.8 (0.7-1.0) | 0.8 (0.7-1.0)* | 343 (0.7) | 0.9 (0.8-1.0) | 0.8 (0.7-1.0)* | 1394 (3.0) | 0.91 (0.85-0.98) | 0.86 (0.80-0.93)** |
| **Smoking Status** |  |  |  |  |  |  |  |  |  |
| No | 340 (0.4) | 1 | 1 | 663 (0.8) | 1 | 1 | 2556 (2.9) | 1 | 1 |
| Yes | 32 (0.6) | 1.6 (1.1-2.4) | 1.2 (0.8-1.7) | 73 (1.4) | 1.9 (1.5-2.4) | 1.3 (1.0-1.7)* | 366 (7.2) | 2.5 (2.2-2.8) | 1.7 (1.6-2.0)** |
| Unknown | 7 (1.3) | 3.4 (1.6-7.1) | 1.7 (0.7-3.9) | 10 (1.9) | 2.5 (1.3-4.6) | 1.6 (0.8-3.1) | 40 (7.4) | 2.6 (1.9-3.5) | 1.8 (1.3-2.4)** |
| **Birthweight (g)** |  |  |  |  |  |  |  |  |  |
| 2000-2499 | 54 (1.3) | 1 | 1 | 105 (2.6) | 1 | 1 | 304 (7.6) | 1 | 1 |
| <1500 | 10 (37.6) | 28.9 (14.6-57.4) | 9.0 (3.9-20.9)** | 13 (48.9) | 19.5 (10.8-35.2) | 8.0 (4.0-16.2)** | 19 (71.4) | 10.1 (6.2-16.3) | 6.6 (3.9-11.1)** |
| 1500-1999 | 32 (13.9) | 10.4 (6.7-16.2) | 5.5 (3.4-8.9)** | 53 (23.0) | 9.0 (6.4-12.5) | 5.8 (4.0-8.3)** | 90 (39.0) | 5.3 (4.2-6.7) | 4.4 (3.4-5.6)** |
| 2500-2999 | 118 (0.5) | 0.4 (0.3-0.5) | 0.5 (0.4-0.7)** | 239 (1.0) | 0.4 (0.3-0.5) | 0.5 (0.4-0.6)** | 950 (4.0) | 0.52 (0.46-0.60) | 0.6 (0.5-0.7)** |
| 3000-3499 | 104 (0.3) | 0.2 (0.1-0.3) | 0.3 (0.2-0.4)** | 214 (0.5) | 0.20 (0.16-0.25) | 0.3 (0.2-0.4)** | 1065 (2.6) | 0.34 (0.30-0.39) | 0.40 (0.35-0.46)** |
| 3500-3999 | 39 (0.2) | 0.14 (0.09-0.22) | 0.2 (0.1-0.3)** | 88 (0.4) | 0.17 (0.13-0.22) | 0.22 (0.16-0.3)** | 434 (2.2) | 0.28 (0.24-0.33) | 0.34 (0.29-0.40)** |
| 4000-4499 | 16 (0.4) | 0.3 (0.2-0.5) | 0.3 (0.2-0.6)** | 26 (0.6) | 0.24 (0.16-0.37) | 0.3 (0.2-0.4)** | 79 (1.9) | 0.25 (0.20-0.32) | 0.3 (0.2-0.4)** |
| 4500-4999 | 5 (0.9) | 0.7 (0.3-1.7) | 0.3 (0.1-0.9)* | 7 (1.3) | 0.5 (0.2-1.0) | 0.3 (0.1-0.6)* | 19 (3.4) | 0.5 (0.3-0.7) | 0.4 (0.3-0.6)** |
| >5000 | 1 (1.3) | 0.9 (0.1-6.8) | 0.4 (0.1-3.2) | 1 (1.3) | 0.5 (0.1-3.5) | 0.2 (0.03-1.7) | 2 (2.5) | 0.3 (0.1-1.3) | 0.3 (0.1-1.0) |
| Unknown | 0 (0) | -- | -- | 0 (0) | -- | -- | 0 (0) | -- | -- |
| **Maternal Education** |  |  |  |  |  |  |  |  |  |
| <8th grade | 13 (0.9) | 1 | 1 | 20 (1.4) | 1 | 1 | 51 (3.5) | 1 | 1 |
| 9-12th grade, no diploma | 54 (0.5) | 0.5 (0.3-1.0) | 0.6 (0.3-1.1) | 139 (1.2) | 0.9 (0.6-1.4) | 0.9 (0.6-1.5) | 597 (5.2) | 1.5 (1.1-2.0) | 1.3 (1.0-1.8) |
| HS or GED | 135 (0.4) | 0.5 (0.3-0.8) | 0.6 (0.3-1.0) | 268 (0.8) | 0.6 (0.4-1.0) | 0.7 (0.4-1.1) | 1157 (3.6) | 1.0 (0.8-1.4) | 1.1 (0.8-1.4) |
| Some college credit | 85 (0.3) | 0.4 (0.2-0.7) | 0.5 (0.3-0.9)* | 162 (0.7) | 0.5 (0.3-0.8) | 0.6 (0.4-0.9)* | 706 (2.9) | 0.8 (0.6-1.1) | 1.0 (0.7-1.3) |
| Associates Degree | 30 (0.4) | 0.5 (0.3-0.9) | 0.6 (0.3-1.1) | 54 (0.8) | 0.6 (0.3-0.9) | 0.7 (0.4-1.2) | 161 (2.3) | 0.7 (0.5-0.9) | 0.9 (0.6-1.2) |
| Bachelors Degree | 32 (0.3) | 0.3 (0.2-0.6) | 0.4 (0.2-0.9)* | 58 (0.5) | 0.4 (0.2-0.7) | 0.6 (0.3-0.9)* | 176 (1.6) | 0.5 (0.3-0.6) | 0.8 (0.6-1.1) |
| Masters Degree | 13 (0.3) | 0.3 (0.2-0.7) | 0.4 (0.2-1.0)* | 27 (0.6) | 0.4 (0.2-0.8) | 0.6 (0.3-1.2) | 59 (1.3) | 0.4 (0.3-0.5) | 0.7 (0.5-1.0) |
| Doctorate/Professional Degree | 4 (0.4) | 0.5 (0.2-1.4) | 0.6 (0.2-2.0) | 4 (0.4) | 0.3 (0.1-0.9) | 0.4 (0.2-1.3) | 10 (1.0) | 0.3 (0.1-0.6) | 0.6 (0.3-1.1) |
| Unknown | 13 (1.7) | 1.9 (0.9-4.1) | 1.7 (0.7-3.9) | 14 (1.8) | 1.3 (0.7-2.7) | 1.2 (0.6-2.5) | 45 (5.9) | 1.7 (1.1-2.5) | 1.6 (1.1-2.4)* |
| **Maternal BMI** |  |  |  |  |  |  |  |  |  |
| Underweight (<18.5) | 17 (0.6) | 1 | 1 | 24 (0.9) | 1 | 1 | 91 (3.3) | 1 | 1 |
| Normal (18.5-24.9) | 106 (0.3) | 0.6 (0.3-1.0) | 0.6 (0.4-1.1) | 214 (0.7) | 0.8 (0.5-1.2) | 0.9 (0.6-1.5) | 861 (2.8) | 0.9 (0.7-1.1) | 1.0 (0.8-1.2) |
| Overweight (25-29.9) | 87 (0.4) | 0.6 (0.4-1.0) | 0.7 (0.4-1.2) | 181 (0.7) | 0.9 (0.6-1.3) | 1.1 (0.7-1.7) | 683 (2.8) | 0.9 (0.7-1.1) | 1.1 (0.9-1.4) |
| Obesity I (30-34.9) | 78 (0.5) | 0.8 (0.5-1.3) | 0.9 (0.5-1.5) | 143 (0.9) | 1.0 (0.7-1.6) | 1.3 (0.8-1.0) | 529 (3.2) | 1.0 (0.8-1.2) | 1.3 (1.0-1.6)* |
| Obesity II (35-39.9) | 29 (0.3) | 0.5 (0.3-1.0) | 0.6 (0.3-1.1) | 66 (0.7) | 0.9 (0.5-1.4) | 1.0 (0.6-1.7) | 299 (3.4) | 1.0 (0.8-1.3) | 1.3 (1.0-1.7)* |
| Obesity III (>40) | 37 (0.5) | 0.8 (0.5-1.4) | 0.8 (0.4-1.4) | 75 (1.0) | 1.2 (0.7-1.8) | 1.3 (0.8-2.1) | 330 (4.4) | 1.3 (1.1-1.7) | 1.7 (1.3-2.1)** |
| Unknown | 25 (0.8) | 1.3 (0.7-2.3) | 1.0 (0.5-1.8) | 43 (1.3) | 1.5 (0.9-2.5) | 1.5 (0.9-2.4) | 169 (5.1) | 1.6 (1.2-2.0) | 1.7 (1.3-2.2)** |
| **Maternal age** |  |  |  |  |  |  |  |  |  |
| 15-19 | 32 (0.5) | 1 | 1 | 69 (1.0) | 1 | 1 | 290 (4.1) | 1 | 1 |
| 20-24 | 101 (0.4) | 0.9 (0.6-1.3) | 1.0 (0.7-1.6) | 213 (0.8) | 0.9 (0.7-1.1) | 1.0 (0.7-1.3) | 997 (3.9) | 1.0 (0.8-1.1) | 0.8 (0.7-1.0)* |
| 25-29 | 101 (0.4) | 0.8 (0.5-1.2) | 1.0 (0.7-1.6) | 200 (0.7) | 0.7 (0.6-1.0) | 0.8 (0.6-1.1) | 852 (3.0) | 0.74 (0.65-0.85) | 0.6 (0.5-0.7)** |
| 30-34 | 66 (0.3) | 0.7 (0.5-1.1) | 1.0 (0.6-1.7) | 140 (0.7) | 0.7 (0.5-1.0) | 0.8 (0.6-1.2) | 478 (2.4) | 0.6 (0.5-0.7) | 0.45 (0.37-0.53)** |
| 35-39 | 48 (0.5) | 1.0 (0.7-1.6) | 1.3 (0.8-2.1) | 79 (0.8) | 0.8 (0.6-1.1) | 0.8 (0.6-1.2) | 241 (2.3) | 0.6 (0.5-0.7) | 0.4 (0.3-0.5)** |
| 40+ | 31 (1.2) | 2.7 (1.7-4.5) | 2.9 (1.6-5.3)** | 45 (1.8) | 1.8 (1.3-2.7) | 1.7 (1.1-2.6)* | 104 (4.1) | 1.0 (0.8-1.3) | 0.7 (0.5-0.9)* |
| **Previous live births** |  |  |  |  |  |  |  |  |  |
| 1 to 2 | 162 (0.4) | 1 | 1 | 329 (0.7) | 1 | 1 | 1393 (3.1) | 1 | 1 |
| None | 150 (0.4) | 1.2 (1.0-1.5) | 0.9 (0.7-1.1) | 260 (0.8) | 1.0 (0.9-1.2) | 0.7 (0.6-0.9)* | 811 (2.4) | 0.76 (0.70-0.83) | 0.58 (0.53-0.64)** |
| 3 to 4 | 39 (0.3) | 0.9 (0.6-1.3) | 0.7 (0.5-1.0)* | 109 (0.9) | 1.2 (1.0-1.5) | 1.0 (0.8-1.3) | 541 (4.5) | 1.4 (1.3-1.6) | 1.4 (1.3-1.6)** |
| 5 or more | 23 (0.7) | 2.0 (1.3-3.1) | 1.2 (0.7-1.9) | 42 (1.3) | 1.8 (1.3-2.5) | 1.3 (0.9-1.8) | 193 (6.0) | 1.9 (1.7-2.2) | 1.9 (1.6-2.2)** |
| Unknown | 5 (1.2) | 3.4 (1.4-8.2) | 1.5 (0.6-3.9) | 6 (1.5) | 2.0 (0.9-4.5) | 1.1 (0.5-2.5) | 24 (5.9) | 1.9 (1.3-2.8) | 1.3 (0.9-2.0) |
| **Gestational age [mean(SD)]** | 38.45 (1.28) | 0.67 (0.61-0.74) | 0.88 (0.90-0.97)* | 38.48 (1.24) | 0.70 (0.65-0.74) | 0.9 (0.8-1.0)* | 38.61 (1.13) | 0.78 (0.76-0.81) | 0.93 (0.90-0.97)** |

*Wald p-value < 0.05; **Wald p-value < 0.001

*OR (95% CI)= Odds ratios and associated 95% confidence intervals; GED=General Educational Development; BMI=Body Mass Index; SD=Standard Deviation*

Odds ratios and 95% confidence intervals were adjusted for infant sex, maternal age, maternal smoking status, infant birthweight, maternal education, maternal BMI, previous number of live births and gestational age.
