# Supplementary material for: Associations between low Apgar scores and mortality by race in the United States: A cohort study of 6,809,653 infants
Source: PLoS Med. 2022 Jul 12;19(7):e1004040. doi: 10.1371/journal.pmed.1004040 (PMC9275714; doi:10.1371/journal.pmed.1004040)
Supplement: S8 Table — (DOCX) [file pmed.1004040.s008.docx]

**Supplementary Table 8: Unadjusted and Adjusted Odds Ratios for Mortality for Multivariable Models in Non-Hispanic Asian Cohort**

|  | **Early Neonatal Mortality (<7 days)** | | | **Overall Neonatal Mortality (<28 days)** | | | **Infant Mortality (<1 year)** | | |
| --- | --- | --- | --- | --- | --- | --- | --- | --- | --- |
|  | Early neonatal mortality [n (deaths per 1,000 births)] | Unadjusted OR (95% CI) | Adjusted OR (95% CI) | Overall neonatal mortality [n (deaths per 1,000 births)] | Unadjusted OR (95% CI) | Adjusted OR (95% CI) | Infant mortality [n (deaths per 1,000 births)] | Unadjusted OR (95% CI) | Adjusted OR (95% CI) |
| **5-Minute Apgar** |  |  |  |  |  |  |  |  |  |
| Normal (7-10) | 51 (0.1) | 1 | 1 | 98 (0.2) | 1 | 1 | 352 (0.8) | 1 | 1 |
| Intermediate (4-6) | 16 (5.5) | 48.4 (27.6-85.0) | 45.8 (25.6-82.0)** | 22 (7.5) | 34.7 (21.8-55.2) | 32.5 (20.2-52.5)** | 38 (13.0) | 16.8 (12.0-23.5) | 16.3 (11.5-23.0)** |
| Low (0-3) | 47 (59.9) | 559.2 (373.8-836.6) | 545.8 (354.6-840.1)** | 56 (71.3) | 351.0 (250.7-491.3) | 335.4 (234.1-480.4)** | 60 (76.4) | 105.2 (79.3-139.7) | 100.4 (74.5-135.4)** |
| **Year of birth** |  |  |  |  |  |  |  |  |  |
| 2016 | 51 (0.2) | 1 | 1 | 88 (0.4) | 1 | 1 | 238 (1.0) | 1 | 1 |
| 2017 | 63 (0.3) | 1.3 (0.9-1.8) | 1.4 (1.0-2.1) | 88 (0.4) | 1.0 (0.8-1.4) | 1.1 (0.8-1.5) | 212 (0.9) | 0.9 (0.8-1.1) | 0.9 (0.8-1.1) |
| **Infant Sex** |  |  |  |  |  |  |  |  |  |
| Male | 63 (0.3) | 1 | 1 | 99 (0.4) | 1 | 1 | 248 (1.1) | 1 | 1 |
| Female | 51 (0.2) | 0.9 (0.6-1.2) | 0.8 (0.6-1.2) | 77 (0.3) | 0.8 (0.6-1.1) | 0.8 (0.6-1.1) | 202 (0.9) | 0.9 (0.7-1.03) | 0.8 (0.7-1.0)* |
| **Smoking Status** |  |  |  |  |  |  |  |  |  |
| No | 113 (0.3) | 1 | 1 | 175 (0.4) | 1 | 1 | 443 (1.0) | 1 | 1 |
| Yes | 0 (0) | -- | -- | 0 (0) | -- | -- | 5 (2.1) | 2.2 (0.9-5.2) | 1.3 (0.5-3.3) |
| Unknown | 1 (1.1) | 4.5 (0.6-32.2) | 1.7 (0.2-18.4) | 1 (1.1) | 2.9 (0.4-20.7) | 1.1 (0.1-11.2) | 2 (2.3) | 2.3 (0.6-9.2) | 1.2 (0.3-5.5) |
| **Birthweight (g)** |  |  |  |  |  |  |  |  |  |
| 2000-2499 | 19 (1.4) | 1 | 1 | 27 (2.0) | 1 | 1 | 59 (4.4) | 1 | 1 |
| <1500 | 3 (49.2) | 36.6 (10.6-127.2) | 22.5 (3.9-132.0)** | 3 (49.2) | 25.8 (7.6-87.3) | 13.1 (2.6-67.3)* | 4 (65.6) | 16.0 (5.6-45.4) | 8.9 (2.5-31.5)** |
| 1500-1999 | 6 (8.9) | 6.4 (2.5-16.0) | 4.5 (1.5-13.3)* | 8 (11.9) | 6.0 (2.7-13.3) | 4.5 (1.8-11.2)* | 11 (16.4) | 3.8 (2.0-7.2) | 2.7 (1.3-5.5)* |
| 2500-2999 | 34 (0.3) | 0.2 (0.1-0.4) | 0.3 (0.2-0.6)** | 49 (0.5) | 0.2 (0.1-0.4) | 0.3 (0.2-0.5)** | 132 (1.3) | 0.3 (0.2-0.4) | 0.3 (0.2-0.5)** |
| 3000-3499 | 32 (0.2) | 0.11 (0.06-0.19) | 0.2 (0.1-0.3)** | 57 (0.3) | 0.14 (0.09-0.22) | 0.2 (0.1-0.3)** | 155 (0.7) | 0.17 (0.13-0.23) | 0.20 (0.15-0.28)** |
| 3500-3999 | 17 (0.2) | 0.12 (0.06-0.23) | 0.2 (0.1-0.4)** | 27 (0.3) | 0.13 (0.08-0.22) | 0.2 (0.1-0.4)** | 77 (0.7) | 0.17 (0.12-0.24) | 0.2 (0.1-0.3)** |
| 4000-4499 | 2 (0.1) | 0.08 (0.02-0.33) | 0.09 (0.02-0.39)* | 2 (0.1) | 0.05 (0.01-0.22) | 0.06 (0.01-0.27)** | 9 (0.5) | 0.11 (0.05-0.22) | 0.11 (0.05-0.23)** |
| 4500-4999 | 1 (0.5) | 0.3 (0.1-2.6) | 0.19 (0.02-1.64) | 2 (1.0) | 0.5 (0.1-2.0) | 0.3 (0.1-1.5) | 2 (1.0) | 0.2 (0.1-0.9) | 0.2 (0.04-0.6)* |
| >5000 | 0 (0) | -- | -- | 1 (4.1) | 2.1 (0.3-15.2) | 0.6 (0.1-5.4) | 1 (4.1) | 0.9 (0.1-6.8) | 0.3 (0.04-2.7) |
| Unknown | 0 (0) | -- | -- | 0 (0) | -- | -- | 0 (0) | -- | -- |
| **Maternal Education** |  |  |  |  |  |  |  |  |  |
| <8th grade | 4 (0.4) | 1 | 1 | 8 (0.7) | 1 | 1 | 22 (1.9) | 1 | 1 |
| 9-12th grade, no diploma | 6 (0.3) | 0.9 (0.3-3.1) | 1.0 (0.3-3.7) | 11 (0.6) | 0.8 (0.3-2.0) | 0.9 (0.4-2.4) | 31 (1.6) | 0.8 (0.5-1.4) | 1.0 (0.5-1.7) |
| HS or GED | 15 (0.3) | 0.8 (0.3-2.4) | 0.9 (0.3-2.9) | 23 (0.4) | 0.6 (0.3-1.4) | 0.7 (0.3-1.6) | 65 (1.2) | 0.6 (0.4-1.0) | 0.7 (0.4-1.2) |
| Some college credit | 12 (0.3) | 0.7 (0.2-2.3) | 0.8 (0.2-2.7) | 16 (0.3) | 0.5 (0.2-1.1) | 0.6 (0.2-1.4) | 63 (1.3) | 0.7 (0.4-1.1) | 0.9 (0.5-1.4) |
| Associates Degree | 4 (0.1) | 0.4 (0.1-1.6) | 0.5 (0.1-2.1) | 8 (0.3) | 0.4 (0.2-1.1) | 0.5 (0.2-1.4) | 23 (0.8) | 0.4 (0.2-0.8) | 0.5 (0.3-1.0)* |
| Bachelors Degree | 43 (0.3) | 0.8 (0.3-2.2) | 1.2 (0.4-3.4) | 65 (0.4) | 0.6 (0.3-1.2) | 0.9 (0.4-1.9) | 152 (1.0) | 0.5 (0.3-0.8) | 0.8 (0.5-1.2) |
| Masters Degree | 20 (0.2) | 0.6 (0.2-1.8) | 0.9 (0.3-2.8) | 30 (0.3) | 0.5 (0.2-1.0) | 0.7 (0.3-1.6) | 59 (0.6) | 0.3 (0.2-0.5) | 0.5 (0.3-0.8)* |
| Doctorate/Professional Degree | 4 (0.1) | 0.3 (0.1-1.3) | 0.6 (0.1-2.4) | 8 (0.2) | 0.3 (0.1-0.9) | 0.6 (0.2-1.6) | 22 (0.6) | 0.3 (0.2-0.6) | 0.6 (0.3-1.01) |
| Unknown | 6 (0.9) | 2.6 (0.7-9.1) | 3.4 (0.9-13.0) | 7 (1.0) | 1.5 (0.5-4.1) | 1.9 (0.7-5.6) | 13 (1.9) | 1.0 (0.5-2.0) | 1.3 (0.6-2.7) |
| **Maternal BMI** |  |  |  |  |  |  |  |  |  |
| Underweight (<18.5) | 9 (0.3) | 1 | 1 | 14 (0.4) | 1 | 1 | 39 (1.1) | 1 | 1 |
| Normal (18.5-24.9) | 54 (0.2) | 0.8 (0.4-1.6) | 0.9 (0.4-1.8) | 88 (0.3) | 0.8 (0.5-1.4) | 0.9 (0.5-1.6) | 235 (0.9) | 0.8 (0.6-1.1) | 0.9 (0.6-1.2) |
| Overweight (25-29.9) | 31 (0.3) | 1.3 (0.6-2.7) | 1.2 (0.5-2.5) | 44 (0.5) | 1.2 (0.6-2.1) | 1.1 (0.6-2.1) | 108 (1.1) | 1.02 (0.7-1.5) | 1.01 (0.7-1.5) |
| Obesity I (30-34.9) | 14 (0.5) | 1.9 (0.8-4.5) | 1.7 (0.7-4.1) | 20 (0.7) | 1.8 (0.9-3.5) | 1.6 (0.8-3.3) | 40 (1.4) | 1.3 (0.8-2.0) | 1.2 (0.8-1.9) |
| Obesity II (35-39.9) | 1 (0.2) | 0.6 (0.1-4.7) | 0.5 (0.1-4.2) | 1 (0.2) | 0.4 (0.05-2.9) | 0.3 (0.04-2.6) | 7 (1.1) | 1.0 (0.4-2.2) | 0.9 (0.4-2.0) |
| Obesity III (>40) | 1 (0.5) | 2.0 (0.3-15.8) | 2.1 (0.3-18.0) | 3 (1.6) | 3.9 (1.1-13.5) | 3.9 (1.1-14.4)* | 7 (3.6) | 3.3 (1.5-7.3) | 2.8 (1.2-6.4)* |
| Unknown | 4 (0.3) | 1.3 (0.4-4.2) | 1.3 (0.4-4.6) | 6 (0.5) | 1.2 (0.5-3.2) | 1.3 (0.5-3.4) | 14 (1.2) | 1.04 (0.6-1.9) | 2.8 (1.2-6.4) |
| **Maternal age** |  |  |  |  |  |  |  |  |  |
| 15-19 | 1 (0.3) | 1 | 1 | 1 (0.3) | 1 | 1 | 3 (0.9) | 1 | 1 |
| 20-24 | 4 (0.1) | 0.4 (0.1-3.7) | 0.4 (0.04-3.6) | 10 (0.3) | 1.0 (0.1-8.1) | 1.1 (0.1-8.8) | 43 (1.4) | 1.5 (0.5-4.8) | 1.6 (0.5-5.2) |
| 25-29 | 37 (0.3) | 1.0 (0.1-7.3) | 1.0 (0.1-7.7) | 54 (0.5) | 1.5 (0.2-10.6) | 1.6 (0.2-12.3) | 127 (1.1) | 1.2 (0.4-3.6) | 1.4 (0.4-4.4) |
| 30-34 | 42 (0.2) | 0.8 (0.1-5.5) | 0.7 (0.1-5.7) | 66 (0.4) | 1.2 (0.2-8.6) | 1.3 (0.2-9.8) | 171 (1.0) | 1.03 (0.3-3.2) | 1.2 (0.4-3.9) |
| 35-39 | 22 (0.2) | 0.7 (0.1-5.4) | 0.6 (0.1-4.9) | 35 (0.4) | 1.2 (0.2-8.4) | 1.1 (0.1-8.5) | 85 (0.9) | 0.9 (0.3-3.0) | 1.0 (0.3-3.3) |
| 40+ | 8 (0.4) | 1.2 (0.2-9.3) | 0.7 (0.1-6.5) | 10 (0.5) | 1.5 (0.2-11.4) | 1.03 (0.1-8.8) | 21 (1.0) | 1.02 (0.3-3.4) | 0.9 (0.3-3.1) |
| **Previous live births** |  |  |  |  |  |  |  |  |  |
| 1 to 2 | 56 (0.3) | 1 | 1 | 86 (0.4) | 1 | 1 | 240 (1.1) | 1 | 1 |
| None | 51 (0.2) | 1.0 (0.7-1.4) | 0.6 (0.4-0.9)* | 77 (0.4) | 1.0 (0.7-1.3) | 0.7 (0.5-0.9)* | 169 (0.8) | 0.8 (0.6-0.9) | 0.6 (0.5-0.7)** |
| 3 to 4 | 5 (0.3) | 1.0 (0.4-2.6) | 0.8 (0.3-2.1) | 9 (0.5) | 1.2 (0.6-2.4) | 1.0 (0.5-2.0) | 26 (1.4) | 1.3 (0.8-1.9) | 1.1 (0.7-1.6) |
| 5 or more | 2 (0.6) | 2.5 (0.6-10.1) | 1.0 (0.2-5.2) | 4 (1.3) | 3.2 (1.3-8.8) | 1.8 (0.6-5.7) | 13 (4.1) | 3.8 (2.2-6.6) | 2.8 (1.5-5.2)** |
| Unknown | 0 (0) | -- | -- | 0 (0) | -- | -- | 2 (2.6) | 2.4 (0.6-9.8) | 2.1 (0.5-8.6) |
| **Gestational age [mean(SD)]** | 38.5 (1.3) | 0.7 (0.6-0.8) | 0.8 (0.7-1.0)* | 38.5 (1.3) | 0.7 (0.6-0.8) | 0.8 (0.7-1.0)* | 38.6 (1.2) | 0.73 (0.67-0.80) | 0.9 (0.8-1.0)* |

*Wald p-value < 0.05; **Wald p-value < 0.001

*OR (95% CI)= Odds ratios and associated 95% confidence intervals; GED=General Educational Development; BMI=Body Mass Index; SD=Standard Deviation*

Odds ratios and 95% confidence intervals were adjusted for infant sex, maternal age, maternal smoking status, infant birthweight, maternal education, maternal BMI, previous number of live births and gestational age.
