# Supplementary material for: Associations between low Apgar scores and mortality by race in the United States: A cohort study of 6,809,653 infants
Source: PLoS Med. 2022 Jul 12;19(7):e1004040. doi: 10.1371/journal.pmed.1004040 (PMC9275714; doi:10.1371/journal.pmed.1004040)
Supplement: S9 Table — (DOCX) [file pmed.1004040.s009.docx]

**Supplementary Table 9: Unadjusted and Adjusted Odds Ratios for Mortality for Multivariable Models in Non-Hispanic Other Cohort**

|  | **Early Neonatal Mortality (<7 days)** | | | **Overall Neonatal Mortality (<28 days)** | | | **Infant Mortality (<1 year)** | | |
| --- | --- | --- | --- | --- | --- | --- | --- | --- | --- |
|  | Early neonatal mortality [n (deaths per 1,000 births)] | Unadjusted OR (95% CI) | Adjusted OR (95% CI) | Overall neonatal mortality [n (deaths per 1,000 births)] | Unadjusted OR (95% CI) | Adjusted OR (95% CI) | Infant mortality [n (deaths per 1,000 births)] | Unadjusted OR (95% CI) | Adjusted OR (95% CI) |
| **5-Minute Apgar** |  |  |  |  |  |  |  |  |  |
| Normal (7-10) | 25 (0.1) | 1 | 1 | 88 (0.4) | 1 | 1 | 572 (0.27%) | 1 | 1 |
| Intermediate (4-6) | 9 (3.7) | 30.7 (14.3-65.8) | 27.0 (12.4-58.5)** | 16 (6.5) | 15.5 (9.1-26.5) | 14.0 (8.1-24.1)** | 29 (1.18%) | 4.3 (3.0-6.3) | 4.1 (2.8-6.0)** |
| Low (0-3) | 41 (58.7) | 522.3 (315.8-863.9) | 414.1 (242.6-707.0)** | 46 (65.9) | 167.7 (116.4-241.5) | 133.02 (89.9-196.9)** | 55 (7.88%) | 31.2 (23.4-41.6) | 26.8 (19.8-36.3)** |
| **Year of birth** |  |  |  |  |  |  |  |  |  |
| 2016 | 40 (0.4) | 1 | 1 | 75 (0.7) | 1 | 1 | 315 (0.30%) | 1 | 1 |
| 2017 | 35 (0.3) | 0.9 (0.6-1.4) | 0.8 (0.5-1.2) | 75 (0.7) | 1.0 (0.7-1.4) | 0.9 (0.7-1.3) | 341 (0.32%) | 1.1 (0.9-1.3) | 1.1 (0.9-1.3) |
| **Infant Sex** |  |  |  |  |  |  |  |  |  |
| Male | 39 (0.4) | 1 | 1 | 79 (0.7) | 1 | 1 | 371 (0.34%) | 1 | 1 |
| Female | 36 (0.3) | 1.0 (0.6-1.5) | 1.2 (0.7-1.9) | 71 (0.7) | 0.9 (0.7-1.3) | 1.0 (0.7-1.4) | 285 (0.27%) | 0.8 (0.7-0.9) | 0.8 (0.7-0.9)** |
| **Smoking Status** |  |  |  |  |  |  |  |  |  |
| No | 62 (0.3) | 1 | 1 | 120 (0.6) | 1 | 1 | 492 (0.27%) | 1 | 1 |
| Yes | 12 (0.5) | 1.4 (0.8-2.6) | 0.9 (0.5-1.8) | 27 (1.1) | 1.6 (1.1-2.5) | 1.1 (0.7-1.8) | 148 (0.58%) | 2.2 (1.8-2.6) | 1.6 (1.3-1.9)** |
| Unknown | 1 (0.6) | 1.9 (0.3-13.4) | 1.4 (0.2-10.9) | 3 (1.9) | 2.9 (0.9-9.1) | 1.8 (0.5-6.5) | 16 (0.99%) | 3.8 (2.3-6.2) | 2.8 (1.7-4.6)** |
| **Birthweight (g)** |  |  |  |  |  |  |  |  |  |
| 2000-2499 | 9 (1.8) | 1 | 1 | 15 (3.0) | 1 | 1 | 44 (0.87%) | 1 | 1 |
| <1500 | 1 (25.0) | 14.5 (1.8-116.9) | 3.1 (0.3-35.5) | 3 (75.0) | 27.4 (7.6-98.6) | 11.3 (2.1-60.0)* | 4 (10.00%) | 12.7 (4.3-37.3) | 7.6 (2.2-25.9)* |
| 1500-1999 | 5 (15.2) | 8.7 (2.9-26.2) | 6.7 (1.7-26.3)* | 5 (15.2) | 5.2 (1.9-14.5) | 3.3 (1.0-10.5)* | 11 (3.35%) | 4.0 (2.03-7.8) | 3.2 (1.6-6.6)* |
| 2500-2999 | 14 (0.4) | 0.2 (0.1-0.5) | 0.4 (0.1-0.9)* | 37 (1.0) | 0.3 (0.2-0.6) | 0.5 (0.2-0.8)* | 159 (0.44%) | 0.5 (0.4-0.7) | 0.6 (0.4-0.8)** |
| 3000-3499 | 16 (0.2) | 0.10 (0.05-0.23) | 0.2 (0.1-0.5)** | 41 (0.5) | 0.2 (0.1-0.3) | 0.2 (0.1-0.5)** | 250 (0.28%) | 0.3 (0.2-0.5) | 0.4 (0.3-0.6)** |
| 3500-3999 | 19 (0.3) | 0.2 (0.1-0.4) | 0.3 (0.1-0.9)* | 34 (0.5) | 0.2 (0.1-0.3) | 0.3 (0.1-0.5)** | 143 (0.23%) | 0.3 (0.2-0.4) | 0.3 (0.2-0.5)** |
| 4000-4499 | 6 (0.4) | 0.2 (0.1-0.6) | 0.4 (0.1-1.3) | 9 (0.5) | 0.2 (0.1-0.4) | 0.3 (0.1-0.6)* | 35 (0.21%) | 0.24 (0.15-0.37) | 0.3 (0.2-0.4)** |
| 4500-4999 | 2 (0.7) | 0.4 (0.1-2.0) | 0.5 (0.1-2.6) | 3 (1.1) | 0.4 (0.1-1.3) | 0.4 (0.1-1.3) | 7 (0.26%) | 0.3 (0.1-0.7) | 0.3 (0.1-0.7)* |
| >5000 | 3 (7.6) | 4.3 (1.2-16.0) | 2.3 (0.5-10.8) | 3 (7.6) | 2.6 (0.8-9.0) | 1.2 (0.3-4.7) | 3 (0.76%) | 0.9 (0.3-2.8) | 0.6 (0.2-1.9) |
| Unknown | 0 (0) | -- | -- | 0 (0) | -- | -- | 0 (0.00%) | -- | -- |
| **Maternal Education** |  |  |  |  |  |  |  |  |  |
| <8th grade | 1 (0.4) | 1 | 1 | 2 (0.8) | 1 | 1 | 13 (0.54%) | 1 | 1 |
| 9-12th grade, no diploma | 17 (0.6) | 1.4 (0.2-10.3) | 1.9 (0.2-18.5) | 37 (1.2) | 1.5 (0.4-6.2) | 1.6 (0.4-7.4) | 162 (0.54%) | 1.0 (0.6-1.8) | 0.9 (0.5-1.7) |
| HS or GED | 20 (0.3) | 0.8 (0.1-5.7) | 1.2 (0.1-11.4) | 40 (0.6) | 0.8 (0.2-3.2) | 1.0 (0.2-4.4) | 216 (0.34%) | 0.6 (0.4-1.1) | 0.7 (0.4-1.2) |
| Some college credit | 17 (0.3) | 0.7 (0.1-5.4) | 1.1 (0.1-10.6) | 38 (0.7) | 0.8 (0.2-3.3) | 1.2 (0.3-5.4) | 169 (0.30%) | 0.5 (0.3-1.0) | 0.7 (0.4-1.3) |
| Associates Degree | 7 (0.4) | 1.0 (0.1-8.1) | 1.6 (0.2-17.5) | 14 (0.8) | 1.0 (0.2-4.4) | 1.5 (0.3-7.4) | 38 (0.23%) | 0.4 (0.2-0.8) | 0.6 (0.3-1.2) |
| Bachelors Degree | 6 (0.2) | 0.5 (0.1-4.3) | 1.1 (0.1-12.1) | 11 (0.4) | 0.5 (0.1-2.1) | 1.0 (0.2-5.0) | 31 (0.11%) | 0.2 (0.1-0.4) | 0.4 (0.2-0.8)* |
| Masters Degree | 3 (0.3) | 0.7 (0.1-6.5) | 1.3 (0.1-16.2) | 3 (0.3) | 0.3 (0.1-2.03) | 0.7 (0.1-4.7) | 13 (0.12%) | 0.2 (0.1-0.5) | 0.5 (0.2-1.1) |
| Doctorate/Professional Degree | 1 (0.3) | 0.7 (0.04-10.9) | 1.1 (0.1-23.9) | 1 (0.3) | 0.3 (0.03-3.8) | 0.7 (0.1-8.2) | 3 (0.09%) | 0.2 (0.04-0.6) | 0.4 (0.1-1.3) |
| Unknown | 3 (2.0) | 4.8 (0.5-45.8) | 4.9 (0.4-64.6) | 4 (2.7) | 3.2 (0.6-17.4) | 2.8 (0.4-17.5) | 11 (0.73%) | 1.3 (0.6-3.01) | 1.4 (0.6-3.1) |
| **Maternal BMI** |  |  |  |  |  |  |  |  |  |
| Underweight (<18.5) | 3 (0.5) | 1 | 1 | 7 (1.1) | 1 | 1 | 20 (0.32%) | 1 | 1.00 |
| Normal (18.5-24.9) | 19 (0.2) | 0.5 (0.2-1.7) | 0.6 (0.2-2.1) | 36 (0.05%) | 0.4 (0.2-0.9) | 0.5 (0.2-1.1)* | 191 (0.24%) | 0.8 (0.5-1.2) | 1.0 (0.6-1.5) |
| Overweight (25-29.9) | 21 (0.4) | 0.8 (0.2-2.7) | 0.8 (0.2-2.8) | 39 (0.07%) | 0.6 (0.3-1.4) | 0.7 (0.3-1.7) | 163 (0.30%) | 0.9 (0.6-1.5) | 0.2 (0.8-1.9) |
| Obesity I (30-34.9) | 11 (0.3) | 0.7 (0.2-2.3) | 0.6 (0.1-2.3) | 25 (0.07%) | 0.6 (0.3-1.5) | 0.7 (0.3-1.6) | 129 (0.37%) | 1.2 (0.7-1.8) | 1.5 (0.9-2.3) |
| Obesity II (35-39.9) | 9 (0.5) | 1.01 (0.3-3.7) | 0.8 (0.2-3.3) | 20 (0.11%) | 1.0 (0.4-2.3) | 1.0 (0.4-2.4) | 78 (0.42%) | 1.3 (0.8-2.1) | 1.6 (1.0-2.7) |
| Obesity III (>40) | 6 (0.4) | 0.9 (0.2-3.7) | 0.5 (0.1-2.5) | 13 (0.10%) | 0.9 (0.3-2.2) | 0.8 (0.3-2.1) | 49 (0.36%) | 1.1 (0.7-1.9) | 1.4 (0.8-2.4) |
| Unknown | 6 (1.2) | 2.6 (0.6-10.3) | 1.9 (0.4-9.1) | 10 (0.20%) | 1.8 (0.7-4.8) | 1.5 (0.5-4.3) | 26 (0.53%) | 1.7 (0.9-3.0) | 1.6 (0.9-2.9) |
| **Maternal age** |  |  |  |  |  |  |  |  |  |
| 15-19 | 7 (0.4) | 1 | 1 | 18 (0.10%) | 1 | 1 | 91 (0.49%) | 1 | 1 |
| 20-24 | 21 (0.4) | 0.9 (0.4-2.2) | 1.5 (0.6-3.9) | 48 (0.08%) | 0.8 (0.5-1.4) | 1.0 (0.5-1.8) | 219 (0.37%) | 0.8 (0.6-1.0) | 0.7 (0.5-0.9)* |
| 25-29 | 20 (0.3) | 0.9 (0.4-2.0) | 1.2 (0.4-3.4) | 37 (0.06%) | 0.6 (0.4-1.1) | 0.6 (0.3-1.2) | 190 (0.31%) | 0.6 (0.5-0.8) | 0.5 (0.4-0.7)** |
| 30-34 | 13 (0.3) | 0.8 (0.3-1.9) | 1.3 (0.4-4.0) | 27 (0.06%) | 0.6 (0.3-1.1) | 0.7 (0.3-1.5) | 102 (0.22%) | 0.5 (0.3-0.6) | 0.4 (0.3-0.6)** |
| 35-39 | 9 (0.4) | 1.1 (0.4-3.0) | 1.7 (0.5-5.6) | 14 (0.06%) | 0.7 (0.3-1.3) | 0.7 (0.3-1.6) | 42 (0.19%) | 0.4 (0.3-0.6) | 0.4 (0.2-0.5)** |
| 40+ | 5 (1.1) | 2.9 (0.9-9.2) | 3.0 (0.7-12.5) | 6 (0.13%) | 1.4 (0.5-3.4) | 1.0 (0.3-3.0) | 12 (0.26%) | 0.5 (0.3-1.0) | 0.4 (0.2-0.8)* |
| **Previous live births** |  |  |  |  |  |  |  |  |  |
| 1 to 2 | 28 (0.3) | 1 | 1 | 63 (0.06%) | 1 | 1 | 300 (0.31%) | 1 | 1 |
| None | 34 (0.4) | 1.5 (0.9-2.4) | 1.1 (0.6-1.9) | 57 (0.07%) | 1.1 (0.8-1.5) | 0.8 (0.5-1.1) | 212 (0.26%) | 0.8 (0.7-1.0) | 0.7 (0.5-0.8)** |
| 3 to 4 | 8 (0.3) | 1.1 (0.5-2.4) | 1.02 (0.4-2.4) | 18 (0.07%) | 1.1 (0.6-1.8) | 1.1 (0.6-1.8) | 105 (0.41%) | 1.3 (1.1-1.7) | 1.3 (1.04-1.7)* |
| 5 or more | 5 (0.7) | 2.5 (1.0-6.5) | 1.2 (0.4-4.0) | 11 (0.16%) | 2.5 (1.3-4.7) | 1.8 (0.9-3.7) | 37 (0.53%) | 1.7 (1.2-2.5) | 1.7 (1.2-2.5)* |
| Unknown | 0 (0) | -- | -- | 1 (0.19%) | 2.9 (0.4-20.8) | 0.8 (0.1-8.6) | 2 (0.37%) | 1.2 (0.3-4.9) | 0.6 (0.1-2.7) |
| **Gestational age [mean(SD)]** | 38.7 (1.4) | 0.8 (0.6-1.0) | 0.9 (0.7-1.1) | 38.73 (1.24) | 0.8 (0.7-0.9) | 1.0 (0.8-1.1) | 38.75 (1.15) | 0.81 (0.76-0.87) | 0.94 (0.87-1.01) |

*Wald p-value < 0.05; **Wald p-value < 0.001

*OR (95% CI)= Odds ratios and associated 95% confidence intervals; GED=General Educational Development; BMI=Body Mass Index; SD=Standard Deviation*

Odds ratios and 95% confidence intervals were adjusted for infant sex, maternal age, maternal smoking status, infant birthweight, maternal education, maternal BMI, previous number of live births and gestational age.
