# Supplementary material for: Associations between low Apgar scores and mortality by race in the United States: A cohort study of 6,809,653 infants
Source: PLoS Med. 2022 Jul 12;19(7):e1004040. doi: 10.1371/journal.pmed.1004040 (PMC9275714; doi:10.1371/journal.pmed.1004040)
Supplement: Attachment — Submitted filename: Response to reviewers.docx [file pmed.1004040.s010.docx]

**RE: Associations between low Apgar scores and mortality by race in the United States: a cohort study of 6,809,653 infants (PMEDICINE-D-21-05026R1)**

*Emma Gillette, James P. Boardman, Clara Calvert, Jeeva John, Sarah J. Stock.*

**Response to Reviewers**

**Requests from the editors:**

- Please revise your title according to PLOS Medicine's style. Your title must be nondeclarative and not a question. It should begin with main concept if possible. Please place the study design in the subtitle (i.e., after a colon), e.g., a retrospective cohort study.
  - **We have amended the title to “Associations between low Apgar scores and mortality by race in the United States: a cohort study of 6,809,653 infants”.**
- Please include line numbers in your next draft.
  - **Amended.**
- Is there a chance you can obtain more recent data from this setting?

**Thank you for the suggestion. The addition of the more recent data is unlikely to change our conclusions, given that this analysis was initially conducted solely on the 2016-2017 cohort (3,443,416 births), and the addition of the 2017-2018 cohort (3,366,237 births) led to no changes in our results or conclusions. There was no change in the recommended use of the Apgar score in the time that the newest dataset has become available, so there is limited reason to think that the results would have changed significantly. The 2018-2019 cohort data became available following the completion of this analysis, so we could include it. However, with the large number of births in the U.S. annually and the complexity of the data, addition of a third cohort of births for analysis would require additional analysis time and result in a significant delay to the publication of results. We estimate that the inclusion of this cohort would require an additional 2 months of analysis time, and the lead researcher has since changed positions, so further analysis is not possible.**

- Please conclude the “Introduction” with a clear description of the study question or hypothesis. The description of the database can be moved to the methods section.
  - **We have included a description of the study question at the end of the introduction as follows:**

***“We hypothesize that the associations between 5-minute Apgar scores and early neonatal, overall neonatal and infant mortality differ by race.”***

- *Abstract*:
- Please ensure that all numbers presented in the abstract are present and identical to numbers presented in the main manuscript text.
  - **Amended, all numbers are identical between the abstract and main text.**
- Please quantify the main results (please present both 95% CIs and p values).
  - **We have added both 95% CIs and p values to the abstract results.**
- In the last sentence of the Abstract Methods and Findings section, please describe the main limitation(s) of the study's methodology.
  - **We have added the following text to the end of the abstract:**

**“*The main limitation was missing data due to using routinely collected data.”***

- At this stage, we ask that you include a short, non-technical Author Summary of your research to make findings accessible to a wide audience that includes both scientists and non-scientists.
  - **We have included an Author Summary as follows:**

***“Why was this study done?***

- ***Apgar scores are commonly used indicators of infants’ well-being at birth, and as predictors of mortality and long term disability.***
- ***Apgar scores have been validated in predominantly White populations.***
- ***The impact of race on the relationship between Apgar score and early neonatal (death within 0-6 days of birth), overall neonatal (death within 0-27 days), and infant mortality (death within 1 year) is unknown.***

***What did the researchers do and find?***

- ***We conducted a cohort study of all singleton, term-birth infants born to mothers over 15 years old, without congenital abnormalities, in the United States in 2017 and 2018 (n=6,809,653).***
- ***Our analyses illustrated that race was associated with the assignment of the Apgar score category and with all categories (early neonatal, overall neonatal, and infant mortality) of mortality.***
- ***There was a stronger association between low Apgar score and all categories of mortality among Non-Hispanic White, Non-Hispanic Asian and Hispanic infants than in Non-Hispanic Black and Non-Hispanic Other infants.***

***What do these findings mean?***

- ***These findings suggest that Apgar scores are less useful for estimating the odds of mortality for Non-Hispanic Black and Non-Hispanic Non-Asian infants than for Non-Hispanic White, Non-Hispanic Other infants.***
- ***Apgar scores are useful predictors of morbidity and mortality; however, their association with mortality is influenced by infant race. Further work to understand which components of the score explain differential associations is needed for developing a scoring system that performs equally well across racial groups.”***
- Did your study have a prospective protocol or analysis plan? Please state this (either way) early in the Methods section
  - **We have added the following statement to the Methods section:**

***“No overall prospective analysis plan was used.”***

- Please add the following statement, or similar, to the Methods: "This study is reported as per the Strengthening the Reporting of Observational Studies in Epidemiology (STROBE) guideline (S1 Checklist)."
  - **The above statement has been added to the end of the Methods section.**
- When completing the checklist, please use section and paragraph numbers, rather than page numbers.
  - **The STROBE checklist has been amended to include section and paragraph numbers.**
- In your statistical analyses, please account for clustering of observations at hospital and county levels. Generalized Estimating Equations (GEE) or hierarchical/ multilevel models, among others, may be useful in this case.

**Thank you very much for this suggestion. We understand the editor’s concerns, however the public use files do not include data on either the facility or the state in which the birth occurred, due to confidentiality protections. Thus, we are not able to account for this in the analysis, nor formally test how much of a difference these adjustments might make. Additionally, given that these are population-based data with no selection criteria on the basis of hospital or county level, we do not think that the main findings are undermined by the lack of clustering at the hospital or county level. We would certainly be concerned about this if the study population only included births from a sample of hospitals, however the study population includes almost all (87%) of births in the U.S. Our approach to the analysis (not accounting for clustering) aligns with published analysis of these data from the CDC. We would expect that the impact of using GEE models with clustering on hospital would have a very small impact on the width of the confidence intervals, and certainly would not change any of our conclusions.**

- Did you adjust for maternal comorbidity or medication use? If not, please consider this or acknowledge the lack of adjustment as a limitation

**Thank you for your comment. We did not adjust for maternal medication use, as this data was not included in the NCHS dataset. Additionally, we did not adjust for maternal comorbidities because the available data included only information on pre-pregnancy and gestational diabetes and hypertension, which are not directly related to the infant’s Apgar score. Other maternal comorbidities may be more directly related to the application of the Apgar score and could have been adjusted for in the models, but data were not available in the NCHS dataset. We have addressed this in the limitations as follows:**

***“A further limitation may have been that models were not adjusted for all maternal comorbidities or medication use, because the data to do so reliably were not available.”***

- Please provide p values in addition to 95% CIs in the main text and tables
  - **We have included p-values alongside the 95% CIs throughout the text and tables.**
- Please define the abbreviations in Tables and Figures e.g, GED, BMI, SD
  - **We have defined the abbreviations in the table and figure captions.**
- Please remove the “Role of the funding source”, “Data sharing”, and “conflict of interest” statement in the methods section. This information is captured in the metadata obtained in the submission form
  - **We have removed these sections.**
- Please remove the “Competing Interests”, “Data availability,” and “Funding” statements at the end of the main text. This information is captured in the metadata obtained in the submission form.
  - **We have removed these sections.**
- *References:*
- Please select the PLOS Medicine reference style in your citation manager. In-text reference call outs should be presented as follows noting the absence of spaces within the square brackets, e.g., "... services [1,2]."
  - **We have amended the references to align with the PLOS Medicine reference style.**
- References should have no more and no less than six names before et al. For references with more than six names, please ensure that et al., is inserted after six names
  - **We have amended the references accordingly.**
- Please ensure that journal name abbreviations consistently match those found in the National Center for Biotechnology Information (NCBI) databases. <https://journals.plos.org/plosmedicine/s/submission-guidelines#loc-references>.
  - **We have amended the references accordingly.**

**Comments from the reviewers:
Reviewer #1:**

1. My comments are mainly related to the test of interactions. By using stratified analysis, the author claims that the association between Apgar scores and mortality varies across racial groups. However, whether the difference in the association between Apgar scores and mortality across racial groups is statistically significant or not has not been tested or presented. In order to acheive this, the author may need to used the adjusted model with interaction beteeen Apgar Score category and racial group. With one racial group as a reference group, ratio of AOR needs to be tested and presented.

**Thank you for the comment. We agree that this is an important result, and is included in the paper (results presented in Table 2), however we have tried to improve the clarity of the description of this test. In the methods, we updated the description to read:**

“~~The model describing the association between Apgar score and mortality included an interaction term to estimate the strength of the evidence for differences in the association between Apgar score and mortality by race group~~. ***We formally assessed whether there was evidence that the association between Apgar score and mortality varied by race group by including an interaction term in the adjusted model in the total population.***”

**And in the results, we write:**

***“There was strong evidence that the association between Apgar score and mortality varied by race group (p<0.001)”.***

**We have also added a statement addressing this interaction to the abstract as follows:**

***“There was strong evidence that this association varied by race (p<0.001)*** *with* *adjusted odds ratios (AORs) of 54.4 (95% CI 49.9-59.4) in Non-Hispanic White, 70.02 (95% CI 60.8-80.7) in Hispanic, 23.3 (95% CI 20.3-26.8) in Non-Hispanic Black, 100.4 (95% CI 74.5-135.4) in Non-Hispanic Asian, and 26.8 (95% CI 19.8-36.3) in Non-Hispanic Other infants****.”***

2. The resoluiton of Figure 2 needs to be increased.

**Thank you for the feedback, we have adjusted the resolution of Figure 2 accordingly.

Reviewer #2:** This study examines assess whether the association between low Apgar score and mortality in infants varies across racial groups.

This is a well written paper, and the analysis appears to be appropriate (having said this I would recommend that the paper is reviewed by a statistical reviewer).

The sample size is of course impressive - but I am afraid that this cannot remedy the other concerns I have with the paper.

A large part of the introduction is making the argument that while Apgar scores have been widely used and are highly predictive of infant mortality, the scores have largely been developed and validated in White populations.  I suspect the authors are speaking about predictive validity - mortality.  If so, this is of course an important issue.  The authors also talk about validating the scores to assess infant status at birth.  This is of course an entirely different matter and would involve a study comparing the gold standard judgement (presumably conducted by a physician) with the scores recorded on a birth chart/card.  The authors even describe how Black infants have been shown to be assigned lower Apgar scores than White infants.  This was reinforced by the later description of how there was a lack of research on the 'racial differences in the applicability of the scores..".

Reading this, I was inclined to conclude that validating Apgar scores (not just predictive validity) or trying to understand these differentials might be the focus of the study.  But this is not the case.  The paper, in my reading simply shows that in a country where Black infants are twice as likely to die as White infants (cited by the authors) that Apgar scores predict this and that Non-Hispanic Black and Non-Hispanic Other groups have higher odds of being assigned a low score.  Without wishing to sound dismissive - how is this interesting?  Is that not exactly what would be expected.

As a result I do not think the paper passes muster in terms of novelty for a public health journal of the stature of PLOS Medicine.  It should definitely be published but perhaps in a specialist journal such as an Obstetric journal.

**We thank the reviewer for their comments. We are in agreement that Black and Non-Hispanic Non-Asian infants have higher baseline rates of mortality in the U.S., and that they are more likely to be assigned low Apgar scores than their White counterparts. However, the aim of our study was not to assess the validity of Apgar scores in different racial groups, and as such we did not set out to assess the accuracy of application of Apgar scores against some other gold standard measurement of neonatal wellbeing. Critically, in the absence of any such gold standard data in this dataset, we cannot expand our analyses to explore this. The aim of this study was to determine, for the first time, whether the association between 5-minute Apgar scores and mortality across the first year of life was different across 5 racial groups using real world data from nearly 7 million births.**

**These findings are novel and important to the field because they demonstrate for the first time that the association between a low 5-minute Apgar score and mortality is different by race, and that the associations are significantly weaker for Black and Non-Hispanic Non-Asian infants. The implications of these findings are substantial, considering how commonly Apgar scores are used both in the U.S. and internationally, and how frequently these scores are used in clinical, epidemiological, social science, research, and policy-making settings.  While we agree it is important to understand why the strength of these associations differ by race, other research methods are required to investigate or determine the causes behind the differential associations demonstrated in our study.**

Reviewer #3: Thank you for the opportunity to review this manuscript. In this paper, the authors examine the association of low Apgar scores and infant mortality across different racial groups using vital statistics data of infants born in the US. The paper finds that the five-minute Apgar score is strongly associated with infant mortality but identified differences in the strength of association between across different racial groups. Specifically, the strength of association between low Apgar score and mortality was reduced in Black and non-Hispanic non- Asian groups suggestive of reduced predictive ability of mortality in these groups.

The authors have examined an important topic that could make a significant contribution to the literature. The study has some significant implications for the interpretation and applicability of Apgar scores and their prediction of mortality across different racial groups.

Overall, this is a good paper but could be improved by being more in-depth in its descriptions and analysis of the findings and the meaning of the results as currently some sections are quite superficial. The methods section could be more detailed and the results section is currently very brief. The authors need to more specifically outline what the findings mean for the application and use of Apgar scores across different racial groups apart from stating that caution should be taken when interpreting the scores. I was expecting to see a section on why was the study done, what do these findings mean etc. to really highlight the key takeaway points - this would be helpful in drawing out the key findings of this paper as well.

**We thank the reviewer for their comments. Edits to the methods, results, and conclusion sections have been made in accordance with the other reviewers’ and editor’s requests and comments, and if there are any further editorial changes to be made we will be happy to address them.**

More attention should be paid to detail as there were repeating text in the manuscript and parts that have not been updated after previous revisions (e.g STROBE statement page numbers)

**Thank you for the comment, we have removed repeating sections, such as the description of the database in the introduction and methods section, and updated the STROBE checklist accordingly.**

I have made some further specific comments below for the authors consideration to revise the manuscript.


Specific comments

ABSTRACT

p2, Methods and findings: The detailed description in the results of how many infants of each race were included and excluded cases is not essential for the abstract. Perhaps you can keep the % and drop the n. I would suggest to review this and focus on presenting the most important results.

**We have removed the number of infants for each race and added to the following sentence to add focus to the important results:**

***“There was strong evidence that this association varied by race (p<0.001) with*** *adjusted odds ratios (AORs) of 54.4 (95% CI 49.9-59.4) in Non-Hispanic White, 70.02 (95% CI 60.8-80.7) in Hispanic, 23.3 (95% CI 20.3-26.8) in Non-Hispanic Black, 100.4 (95% CI 74.5-135.4) in Non-Hispanic Asian, and 26.8 (95% CI 19.8-36.3) in Non-Hispanic Other infants.”*

INTRODUCTION
p3, para 1-2: I would recommend the text in paragraph 2 of the introduction be moved up into paragraph 1 (after the first sentence) and the detail about the Apgar score from the second sentence be moved down into a separate paragraph. This is so the problem your study is investigating is upfront. The detail of the components of Apgar score is not so important that it needs to be in the first paragraph.

**We have moved the information from paragraph 2 to follow the first sentence in paragraph 1, and moved the details about the Apgar score to the 2^nd^ paragraph.**

p4, para 2: missing word - "and" before "discrimination"

**Inserted the word “and”.**

p5, para 1: The last sentence of the introduction ("The database consists of…") is not essential here and would be more appropriate in the methods section where a description of the data source is provided.

**Moved the last sentence of the introduction to the data sources section of the methods.**

METHODS
The ethics sub-section can be placed towards the end of the methods section. The ethics statement is also repeated in the Study Population section at the beginning of page 4. This can be removed.

**Removed the ethics statement from the Study Population methods into a separate ethics subsection within methods.**

The methods section does not follow the STROBE checklist and the page numbers in the checklist do not match the current manuscript (e.g there is no Study Design section in the methods but the checklist in the Appendix indicates it is on pages 8-11 in the manuscript). This should be the first section or combined with study population. I would suggest the authors structure the methods section using similar headings to the strobe statement. The "Database" sub-heading should be "Data sources" for example.
Also, the current STROBE statement checklist is missing the first column that indicates which section of the manuscript the information can be found.

**The methods section was updated according to STROBE checklist section titles, and the STROBE checklist was updated with page and section numbers.**

p7, para 2: Outcomes, exposures, covariates - this section needs to be better organised. I would suggest describing the outcome variable first followed by the explanatory variables.

**The “Variables” (re-titled according to the point above) section was re-organized to first explain the outcome variables.**

p8, top paragraph: There is a bit of a mix up or description of analysis and variables in this section as well - the part on what was adjusted for should be in the analysis section but the definition of the variables should remain in this section. Handling of missing values also belongs in the analysis section.

**We moved the description of what models were adjusted for and the handling of the missing data from the variables sub-section to the analysis sub-section.**

Figure 1: please improve the quality of the flow diagram (font is currently blurred) and consider reducing amount of text and perhaps include the use of boxes.

**We have updated Figure 1 to include boxes around the text, reduced the amount of text, and improved the image resolution.**

RESULTS

p10, para 1:  All of the numbers and % in the table do not need to be repeated in the text. Suggest to mention the % for race without the numbers in the text.

**We have updated the results to remove the numbers for each group.**

On the other hand, on p13 there is no description of the magnitude of the odds described at all. Instead of stating that there is only a lower or higher odds, the authors can be more specific - for example,  "…non-Hispanic black infants had almost two times higher odds of being assigned a low Apgar score…" Otherwise the information in the text is not very informative.

**We have updated the text to describe the magnitude of the differences in the odds between the groups:**

***“Compared with Non-Hispanic White infants, Non-Hispanic Black infants had 1.7 times the odds of being assigned a low Apgar score (95% confidence interval [CI]=1.6-1.8) and Non-Hispanic Other infants had 1.3 times the odds (95% CI=1.2-1.4) (Table 2).***”

p15-16. Please indicate which table the statement made in the third sentence in paragraph 1 is located.

**We have updated the sentence to describe that the data was derived from Supplementary Table 3.**

There is no need to describe the chi-square test here - this belongs in the methods/analysis section.

**We removed the mention of the chi-square test in the results and added a description of the test to the methods as follows:**

***“Additionally, we conducted a chi-square test for trend to determine whether there were trends between Apgar score category and early neonatal, overall neonatal and infant mortality rates among each race group.”***

The main results/tables of your study are in the Appendix - I would suggest to move (some) of these to the main manuscript. The unadjusted analyses I would think were less important and could be in the appendix.

**Thank you for the comment, we agree with this suggestion and have moved Supplementary Table 4 (“Adjusted odds ratios for early neonatal mortality, neonatal mortality, and infant mortality in relation to Apgar score category, stratified by maternal race group”) to the main manuscript as Table 3. The previous Table 3 (“Unadjusted odds ratios for early neonatal, overall neonatal and infant mortality by race group”) was moved to the supplementary material as Supplementary Table 4.**

Overall, the description of your results is very brief given the amount of results and tables included.

**Thank you for the comment. Edits to the results section have been made in accordance with the reviewers’ and editor’s requests and comments, and if there are any further editorial changes to be made we will be happy to address them.**

Tables S6-S8 in the Appendix need to be fit to the page as the last columns are cut off. Suggest to include the word documents with the original tables rather than an image.

**Thank you for the suggestion, we included tables and figures as a separate document for this revision.**

DISCUSSION

p16, first para: The first paragraph of a discussion should highlight the most important findngs of the paper. However the authors describe the unadjusted results.
Please also avoid repeating results but rather explaining or interpreting the data in the discussion

**We have edited the discussion section to include a description of the most important findings earlier in the section.**

Reviewer #4: Thanks for allowing me to review your very interesting manuscript. I have several comments, and some questions which may lead to some revisions.

1. I am curious as to why you excluded births to mothers under the age of 15? Whilst the numbers are likely to be small they are a high-risk group, more likely to have births of compromised babies due to fetal growth restriction and other problems of placental dysfunction. I note that you did include women who did not complete 8th grade at school and if this group is included then perhaps the very young should also be there. Could you elaborate on your reasons for their exclusion?

**We excluded mothers younger than 15 years old because there were very small numbers of such mothers included in the dataset (for example, only 25 mothers <15 among the Non-Hispanic Asian group), and we could not have adjusted for this group separately in the models due to the small population size but were concerned about combining these with the 15-19 years olds, where the risk profiles might be quite different.**

2. Similarly, the inclusion of births up to 44 + 6 weeks of gestation is unusual as this might imply a problem of data accuracy. Even without intervention, which is almost universal now before 42 + 6 weeks, very few pregnancies would progress beyond 43 weeks and certainly not beyond 44. Why such a high figure?

**Thank you for the question, we included births up to 44^+6^ weeks because although births at this gestational age are very rare, this was the upper limit included by CDC in the NCHS database, and as such we abided by the database guideline limits.**

3. What is the origin of the list of congenital abnormalities that have been excluded? It contains many obvious severe conditions (e.g., anencephaly) but also some others which are quite common and not really associated with infant mortality (e.g., hypospadias).

**Thank you for the question. Although some abnormalities listed are not directly associated with mortality, we included all conditions that were listed by the CDC as congenital abnormalities for the data accessed from the National Center for Health Statistics. While it may have been reasonable to include infants with some isolated congenital abnormalities, we took a conservative approach by excluding the entire list because minor abnormalities (such as hypospadias) are often associated with a broader phenotype, which may not be captured in the database. For example, while hypospadias is not directly associated with infant mortality, it has been described in many other syndromes, some of which do more directly affect mortality.**

4. I noted that the explanation about the exemption from full ethics review is repeated in the sections on Ethics and Study Population which is a duplication.

**The explanation was removed from the Study Population section.**

5. The overall smoking rates are much lower than I would have expected (6.8%). In my own country (Australia) we are still seeing rates of 10-15% overall, and these vary a lot between different ethnic groups. Can you comment on this, and if you can validate this figure against any other data source? As it is a risk factor for both neonatal and infant mortality it is important to get this right in any analysis, especially when calculation the adjusted OR.

**Thank you for your comment. A number of recent articles have published similar tobacco smoking rates in the United States, including a study by Buyun Liu et al., “Maternal cigarette smoking before and during pregnancy and the risk of preterm birth: A dose-response analysis of 25 million mother-infant pairs”, published in PLOS medicine in 2020 (**https://journals.plos.org/plosmedicine/article?id=10.1371/journal.pmed.1003158)**.**

6. Finally, I am curious as to the predictive value of the Apgar scores for both neonatal mortality (the original use) and later infant mortality. Whilst some causes of infant mortality are linked to conditions which have their origins in the perinatal period many others are not necessarily linked (e.g. trauma, drowning etc). Have you looked separately at the infant deaths which occur beyond 28 days (about 50% of the total cohort of deaths), rather than including all deaths in the infant mortality numbers, which includes the early and late neonatal ones

**Thank you for the comment. We agree that Apgar scores are useful for their strong associations with neonatal mortality, and we have investigated here whether these associations are differential by race for early neonatal, overall neonatal and infant mortality. Because the odds of mortality are additive over time and we assessed these odds across three periods within the first year of life, it was not necessary to illustrate the odds of mortality between the neonatal and infant periods.**

Reviewer #5: Thank you for the opportunity to review this manuscript.  Overall the analyses are well conducted and the manuscript well written.  I have a few minor edits to suggest:

1. The following text should be combined with the Inclusion and Exclusion Criteria to make a single continuous paragraph on study population, which also means that the gestational age criteria do not need to be stated twice:
"Study Population
This population cohort study evaluated singleton infants born between 37+0 and 44+6 weeks between January 1, 2016 and December 31, 2017 in the United States."

**We have combined the inclusion/exclusion criteria paragraph and the study population paragraph reduce redundancy between these sections.**

2. The 2nd sentence of the Study Population paragraph should be added to the Database paragraph: "All data were nonidentifiable and publicly available through the NCHS Division of Vital Statistics and complied with the NCHS, Centers for Disease Control and Prevention (CDC) Data User 6
Agreement Terms and Conditions.23,24"

**We have moved the sentence from the study population section to the data sources section.**

3. The rest of the Study Population paragraph is an exact repeat from the Ethics paragraph and should be deleted, except the last sentence on Strobe reporting guidelines which can be moved to the end of the methods section.

**We have moved the ethics portion of the study population section to a separate ethics section, and included the STROBE reporting guidelines statement at the end of the methods section.**

4. I did not quite follow the logic for the following: "the findings suggest that the odds of mortality based on a low Apgar score are underestimated in these groups" and suggest this statement be deleted.  The remainder of the discussion and conclusion are much clearer in their interpretations.

**We have clarified this statement as follows:**

***“These findings indicate that low Apgar scores are differentially associated with mortality across race groups and, more specifically, suggest that low Apgar scores are less good at discriminating the risk of mortality in Non-Hispanic Black and Non-Hispanic Other infants compared to other race groups.”.***
