# Supplementary material for: Associations between low Apgar scores and mortality by race in the United States: A cohort study of 6,809,653 infants
Source: PLoS Med. 2022 Jul 12;19(7):e1004040. doi: 10.1371/journal.pmed.1004040 (PMC9275714; doi:10.1371/journal.pmed.1004040)
Supplement: Attachment — Submitted filename: PLOS Med Revision Responses II.docx [file pmed.1004040.s011.docx]

**Dr. Sarah J. Stock**

Wellcome Trust Clinical Career Development Fellow

Reader and Honorary Consultant Maternal and Fetal Medicine

Usher Institute, University of Edinburgh

NINE Edinburgh BioQuarter,

9 Little France Road,

Edinburgh EH16 4UX

[Sarah.stock@ed.ac.uk](mailto:Sarah.stock@ed.ac.uk) | +44 (0)7894629934

15 May, 2022

Dear Dr. Odeny,

Thank you for inviting us to revise and submit our manuscript “Associations between low Apgar scores and mortality by race in the United States: a cohort study of 6,809,653 infants” (PMEDICINE-D-21-05026R1) for consideration with PLOS Medicine. We thank the editor and reviewers for their comments, and have addressed the specific points raised in the comments below.

Requests from Editors:
1)      Abstract:
a)      Please structure your abstract using the PLOS Medicine headings (Background, Methods and Findings, Conclusions).
b)      Please combine the Methods and Findings sections into one section, “Methods and findings”
c)      In the last sentence of the Abstract Methods and Findings section, please describe the main limitation(s) of the study's methodology

**We thank the editor for these comments, and have combined the methods and findings sections, and moved the sentence describing the main limitation (“The main limitation was missing data for some variables due to using routinely collected data”) to the end of the combined section.**

Comments from Reviewers:

Reviewer #1: Thank you for addressing reviewers' comments and improving the manuscript. My questions in the previous round has been suffuciently answered.

1. In Table 2 and Table 3, why there's only one p-value for multiple tests for interaction? For instance, in Table 2, there should be seperate p-values for Non-Hispanic white vs Hispanic, and Non-Hispanic white vs Non-hispanic black. In table 3, there should be p-value for normal vs intermedaite comparison, and p-value for normal vs low comparison.

**As requested by the reviewer, we have added p-values which look at the strength of evidence for each subgroup in Tables 2 & 3, replacing the p-values for whether there is evidence overall for an association between the exposure and outcome.**

2. According to the Office of Management and Budget Standars for the Classification of Federal Data on Race and Ethnicity, Hispanic ethnicity is recorded as distinct from race. In this paper, the author used different category and treated Hispanic as a mutually exclusive racial group from other racial groups.  Explanation is needed for the selction of the categories for the analysis.

**Thank you for this comment. While we recognize OMB standards, in this analysis, we utilized single-race categories proposed by the NCHS and detailed in the guidelines for the use of this public-use dataset. Although parents can report more than one race or ethnicity on birth certificates, the NCHS maintains single-race documentation in their datasets in order to bridge current data with older iterations of these datasets. We’ve addressed this in the manuscript in lines 216-218 by adding the sentence:**

**“*This analysis considered Hispanic as a separate race/ethnicity category in accordance with NCHS guidelines for this dataset, which utilize single-race categorizations.”***

Reviewer #2: The authors have responded to reviewer comments


Reviewer #3: Thank you to the authors for their efforts to address the comments and revise the manuscript. The revisions made have improved the manuscript somewhat but I'm afraid there remain some substantial issues which need to be addressed particularly in relation to the methods section and discussion. I have outlined these below:

Minor revisions

ABSTRACT

Line 45 - Stating the limitations is not needed in the abstract

**Thank you for your feedback. The sentence describing the study limitations was added in the recent revisions at the request of the editor.**

Line 49 (findings) - In the sentence, "A total of 6, 728,829 infants…" the percentages should be included after the numbers for easier interpretation. It's not clear why they were removed from the previous version.

**Amended, added percentages to these figures so the sentence now reads:**

***“A total of 6,728,829 (98.8%) infants had normal scores, 63,467 (0.9%) had intermediate scores, and 17,357 (0.3%) had low Apgar scores.”***

Line 52 - abbreviations (AOR) are usually not used in an abstract - please check with journal requirements. As it is not used multiple times in the abstract it may not be necessary.

**Amended, abbreviation was removed from the abstract.**

AUTHORS' SUMMARY

This is a very nice overall summary. Just one small suggestion:
Line 90 - please remove the "n" as it doesn't add anything here and it is stated clearly in the title of the paper.
**Amended, n value was removed.**
Major revisions

METHODS

The methods section needs reorganisation - grouping study design, setting and participants is not following the STROBE guidelines and these are all very different topics, especially participants (study setting may not be needed). This section now also discusses the exclusion and inclusion criteria.
There are now two sections with the heading of "variables" one called "quantitative variables" - all Generally, there should be description of the variables - dependent and independent. The statistical analysis should be separate. Please closely review the STROBE guidelines and other similar papers for order and content of the methods section.
I do not have statistical expertise but is this multivariable regression not multivariate?

Line 213 (Statistical methods and Quantitative Variables) - Please separate out this section into paragraphs as it is too long as one piece of text.

Line 251 - this statement on STROBE should be at the beginning of the methods not in the Ethics statement.

**Thank you for these comments. We have amended the sections by creating a separate “Participants” section, as well as separate “Statistical Methods” and “Variables” sections. The STROBE statement was moved to the first section of the methods, under “Study Design and Setting” (lines 172-174). We have amended the manuscript to include the term “multivariable” everywhere where “multivariate” was previously used.**

RESULTS

All of the figures are still very low quality and not clear.

**Thank you for the feedback – the figures were edited and confirmed with PLOS Medicine’s PACE system to meet the figure requirements.**

Table 1 - this table is very hard to read and has no lines - please format and add some spacing between the variables.

**Amended, we added lines and appropriate spacing to Table 1.**

DISCUSSION

There seems to be a disconnect between the first paragraph of the discussion and the author's summary around the implications of the findings. The discussion is very positive and supportive of the Apgar score while the Authors' summary is more critical indicating that Apgar scores are less useful among non-White populations. The overall conclusions should be based on the results only and need to be consistently communicated throughout the paper.

In my previous review I indicated that the authors should avoid only restating the results in the discussion but instead elaborate on what their results mean. Instead of doing this the text was just removed. The discussion cannot consist only of a summary and implications. The results need to be discussed more adequately and critically.

**Thank you for this comment. In the discussion we discuss the findings in the context of current literature, and discuss strengths, limitations and implications of our findings. We have added the following sentence to the discussion section [lines 407-409] to provide further context in the discussion:**

***“These results add to a body of literature which suggests that the performance of mortality prediction tools in neonatal groups can be influenced by race, as demonstrated by the inclusion of race in the use of an estimator tool for bronchopulmonary dysplasia in preterm infants.”***

**We have also added the following sentence to the conclusion section [lines 466-469]:**

*“****This suggests that while Apgar scores should continue to be used in clinical and research settings, practitioners and researchers should be aware that both the assignment and predictive ability of the Apgar score varies across racial groups.”***
