# Supplementary material for: Associations between low Apgar scores and mortality by race in the United States: A cohort study of 6,809,653 infants
Source: PLoS Med. 2022 Jul 12;19(7):e1004040. doi: 10.1371/journal.pmed.1004040 (PMC9275714; doi:10.1371/journal.pmed.1004040)
Supplement: Attachment — Submitted filename: PLOS Med Revision Responses III.docx [file pmed.1004040.s012.docx]

**Dr. Sarah J. Stock**

Wellcome Trust Clinical Career Development Fellow

Reader and Honorary Consultant Maternal and Fetal Medicine

Usher Institute, University of Edinburgh

NINE Edinburgh BioQuarter,

9 Little France Road,

Edinburgh EH16 4UX

[Sarah.stock@ed.ac.uk](mailto:Sarah.stock@ed.ac.uk) | +44 (0)7894629934

31 May, 2022

Dear Dr. Odeny,

Thank you for inviting us to revise and submit our manuscript “Associations between low Apgar scores and mortality by race in the United States: a cohort study of 6,809,653 infants” (PMEDICINE-D-21-05026R1) for consideration with PLOS Medicine. We thank the editor and reviewers for their comments, and have addressed the specific points raised in the comments below.

Requests from Editors:

1. Abstract: please revise the subheading "Interpretation" to "Conclusion" in line with PLOS Medicine's style
2. Discussion section: please delete the subheadings including "Findings in context", "Implications" and "Strengths and Limitations"

**We thank the editor for these comments, and have accordingly revised the subheading “Interpretation” to “Conclusion” [line 55] and removed the subheadings in the discussion section [lines 385-454].**

Comments from Reviewers:

Reviewer #3: Thank you to the authors for the revisions to the manuscript. The paper is now much clearer and flows well. I have no further comments on this version.
I look forward to seeing this published.

**We thank the reviewer for their feedback.**

Sincerely,

Dr. Sarah J. Stock, on behalf of co-authors
